# Synthesis, Physicochemical Properties and Anti-Fungal Activities of New *Meso*-Arylporphyrins

**DOI:** 10.3390/ijms26051991

**Published:** 2025-02-25

**Authors:** Hayfa Mkacher, Raja Chaâbane-Banaoues, Soukaina Hrichi, Philippe Arnoux, Hamouda Babba, Céline Frochot, Habib Nasri, Samir Acherar

**Affiliations:** 1 Laboratory of Physical Chemistry of Materials, Faculty of Science of Monastir, University of Monastir, Avenue of Environment, Monastir 5019, Tunisia; mkacherhayfa@gmail.com (H.M.); soukaina.hrichi@gmail.com (S.H.); 2 Université de Lorraine, CNRS, LCPM, F-54000 Nancy, France; 3 Laboratory of Medical and Molecular Parasitology-Mycology (LP3M), Faculty of Pharmacy, University of Monastir, LR12ES08, Monastir 5000, Tunisia; rajachaabanebanaoues@gmail.com (R.C.-B.); hamouda.babba@ms.tn (H.B.); 4 Université de Lorraine, CNRS, LRGP, F-54000 Nancy, France; philippe.arnoux@univ-lorraine.fr (P.A.); celine.frochot@univ-lorraine.fr (C.F.)

**Keywords:** *meso*-arylporphyrins, photophysical properties, anti-fungal activities, candida species, dermatophyte species, molecular docking

## Abstract

In this work, we describe the synthesis of three new *meso*-arylporphyrins, named *meso*-tetrakis [4-(nicotinoyloxy)phenyl] porphyrin (**H_2_TNPP**), *meso*-tetrakis [4-(picolinoyloxy)phenyl] porphyrin (**H_2_TPPP**), and *meso*-tetrakis [4-(isonicotinoyloxy) phenyl] porphyrin (**H_2_TIPP**). These new synthesized *meso*-arylporphyrins are characterized using spectroscopic analysis: Fourier Transform Infrared Spectroscopy (FTIR) and One-dimensional Nuclear Magnetic Resonance (1D NMR), and mass spectrometry (MS). The photophysical studies (UV–visible absorption, singlet oxygen (^1^O_2_) luminescence, and fluorescence emissions) demonstrate their potential uses as photosensitizers (PSs) in photodynamic therapy (PDT) applications. An in vitro investigation of the anti-fungal activity of **H_2_TNPP**, **H_2_TPPP**, and **H_2_TIPP** against *Candida* (*C.*) species (*C. albicans*, *C. glabrata*, and *C. tropicalis*) reveals that their minimum inhibitory concentration (MIC) values ranged from 1.25 to 5 mg/mL. In addition, their in vitro anti-fungal susceptibilities against three dermatophyte clinical isolates (*Trichophyton rubrum*, *Microsporum canis*, and *Trichophyton mentagrophytes*) are also evaluated and they demonstrate good anti-fungal activities. A molecular docking study of these *meso*-arylporphyrins as anti-fungal agents against *C. tropicalis* extracellular aspartic proteinases, Protein data Bank in Europe (PDBe code: 1J71) and *Trichophyton rubrum* Sialidases (PDBe code: 7P1D) underlines the possible interactions of **H_2_TNPP**, **H_2_TPPP**, and **H_2_TIPP** with the key amino acid residues of these fungal target proteins.

## 1. Introduction

Human fungal infections cause more than 1.5 million deaths each year, with *Candida* bloodstream infections or invasive candidiasis accounting for 63.6% of patient deaths [1]. The overexposure of fungi to anti-fungal drugs due to an increase in the number of immunocompromised patients, associated with the lack of an effective therapeutic arsenal against fungal infections, has led to the exacerbation of anti-fungal resistance (AFR). AFR is a subset of anti-microbial resistance (AMR), which was recognized in 2021 by the World Health Organization (WHO) as one of the 10 greatest global public health threats facing humanity [2]. If no action is taken, the number of AMR deaths and the cumulative cost could balloon to 10 million deaths and 100 trillion USD per year by 2050 [3]. Thus, AMR represents a significant cost to the world economy, as apart from death and disability, it leads to longer hospital stays (i.e., prolonged illness) and an increased financial burden (i.e., expensive medicines) to those impacted [4,5,6]. The benefits of modern medicine in treating infections (e.g., major surgery and cancer chemotherapy) will be jeopardized without the existence of effective anti-microbial treatments. More specifically, concerning fungi, the WHO, in 2022, published a fungal pathogens priority list to assist researchers in finding solutions to tackle the growing problem of fungal infections and AFR [7], paying more attention to the critical group including *Cryptococcus neoformans*, *Candida auris*, *Aspergillus fumigatus* (*A. fumigatus*), and *Candida albicans* (*C. albicans*).

In recent years, the incidence of iatrogenic invasive fungal infections has been increasing due to the increased number of aplastic chemotherapies, the increasing use of these therapies for autoimmune diseases, transplantation, and the multiplication of invasive acts, with *Candida* spp. and *A. fumigatus* as the main agents, followed by Non-Fumigatus Aspergilli, *Fusarium*, *Pseudoallescheria*, and other opportunistic Ascomycetous fungal pathogens [8,9].

The threat of invasive fungal infections is continually increasing, leading to high morbidity and mortality. This finding prompts researchers to find new drug candidates to fight human pathogenic fungi [10].

As important heterocyclic compounds [11,12], porphyrins and related tetrapyrrolic macrocyclic pigments are notable for their extensive applications in both chemical and biological contexts, particularly their antioxidant activities and interaction with cell membrane compounds [13,14]. The diverse biological functions they exhibit, along with their exceptional photochemical and photophysical properties, are pivotal in fostering interest in these compounds.

Porphyrins can be different due to bridging groups, substituents in the *meso*-positions of the macrocycle, and the closure of neighboring pyrrole substituents into rings [15,16,17,18].

This study presents the synthesis and characterization of three novel *meso*-arylporphyrins. The investigation includes an analysis of the photophysical and electrochemical properties associated with these newly developed new *meso*-arylporphyrins, as well as their in vitro anti-fungal activities (anti-candidal and anti-dermatophyte).

The yeasts *C. albicans*, *Candida glabrata* (*C. glabrata*), and *Candida tropicalis* (*C. tropicalis*) were selected due to their high involvement in human clinical cases. These species are reported to cause both invasive and superficial candidiasis, with *C. albicans* as the predominant species, and increasing resistance to Fluconazole, Echinocaldin, and Caspofungin has been demonstrated [19]. In contrast, dermatophytes are keratinophilic fungi responsible for infections of external tissues, such as onychomycosis and skin lesions. Among them, *Trichophyton rubrum* (*T. rubrum*), *Trichophyton mentagrophytes* (*T. mentagrophytes*), and *Microsporum canis* (*M. canis*) are the main causative agents with recurrence. Terbinafine and Azole resistance have been detected in 30% and 19% of dermatophyte isolates, respectively [20,21].

As a widely recognized technique, molecular docking analysis plays a significant role in facilitating the discovery of novel ligands for proteins whose structures are already established [22]. Therefore, through this study, we assessed the possibility of non-covalent interactions between our new *meso*-arylporphyrins, and the amino acid components found at the potential target proteins: extracellular aspartic proteinases (Protein data Bank in Europe (PDBe) code: 1J71) and Sialidase (KDNase) (PDBe code: 7P1D) of the investigated strains *C. tropicalis* and *T. rubrum*, respectively, which are widely reported to be involved in biological activities [23].

## 2. Results and Discussion

### 2.1. Synthesis

**Figure 1:**
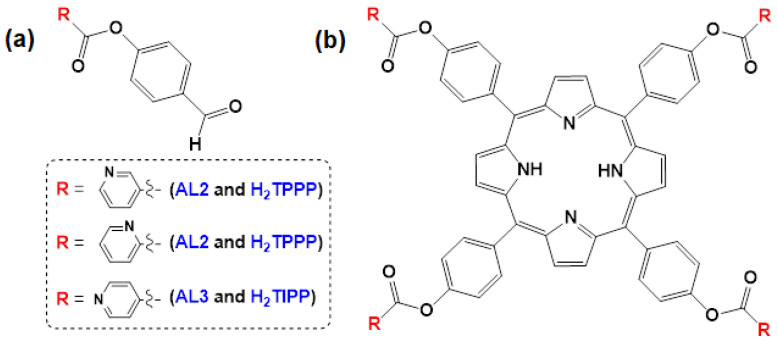
Chemical structures of the three new aldehydes **AL1**, **AL2**, and **AL3** (**a**) and *meso*-arylporphyrins **H_2_TNPP**, **H_2_TPPP**, and **H_2_TIPP** (**b**).

**Scheme 1:**
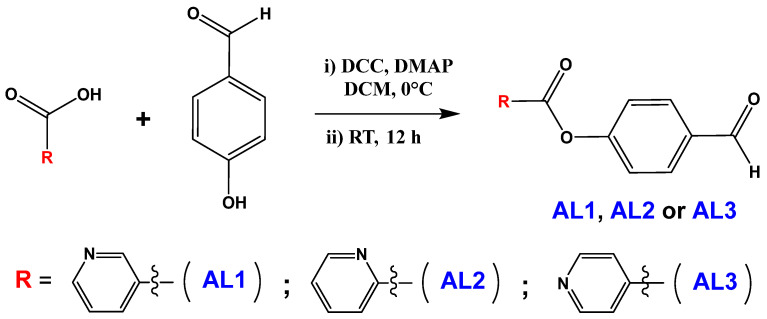
Main chemical preparation steps for aldehydes **AL1**, **AL2**, and **AL3**.

**Scheme 2:**
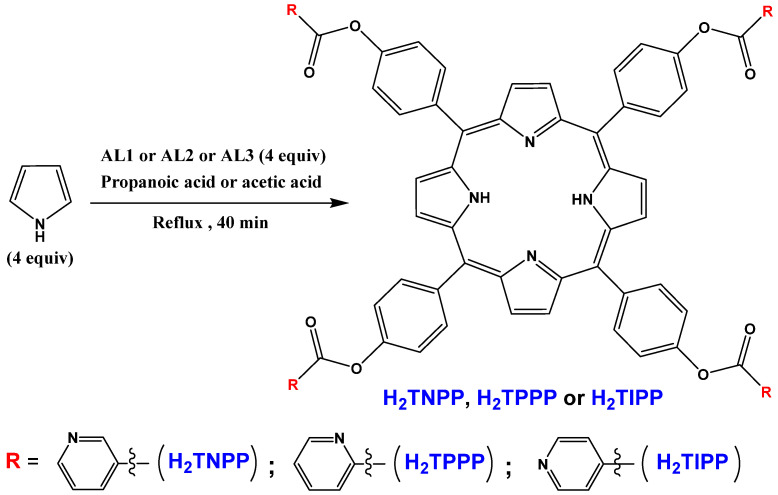
Main chemical preparation steps of *meso*-arylporphyrins **H_2_TNPP**, **H_2_TPPP**, and **H_2_TIPP**.

A two-step synthesis was employed to create the novel *meso*-arylporphyrins, and the synthetic route for the desired compounds is detailed in Section 3.3.1 and Section 3.4.1. Scheme 1 and Scheme 2 In the first step, the new aldehydes were prepared using an esterification reaction via the condensation of a pyridinecarboxylic acid derivative (picolinic, nicotinic or isonicotinic acid) with 4-hydroxybenzaldehyde (Figure 1a) [17]. Then, three new *meso*-arylporphyrins were obtained using the Adler—Longo synthesis technique (Figure 1b) [24]. The structure of each compound was confirmed by Fourier Transform InfraRed (FTIR), ^1^H- and ^13^C- Nuclear Magnetic Resonance (NMR), and elemental analysis.

### 2.2. Characterization

#### 2.2.1. Proton (^1^H) and Carbon (^13^C) J-Modulated Spin-Echo (JMOD) NMR Spectroscopy

The ^1^H and ^13^C NMR spectra of the **AL1**, **AL2**, and **AL3** aldehydes and the *meso*-arylporphyrins **H_2_TNPP**, **H_2_TPPP**, and **H_2_TIPP** are found in Figures S1–S12 (see Supporting Information).

For **AL1**, **AL2**, and **AL3**, the identification of a singlet in the vicinity of 10 ppm in the ^1^H NMR spectra and around 192 ppm in the ^13^C-JMOD NMR spectra corroborates the existence of the aldehyde functional group. Also, ester function is highlighted by the appearance of a signal at around 163 ppm (C=O) and around 155 ppm (C-O) in the ^13^C-JMOD NMR spectra. Analysis of the ^1^H NMR spectra of the three *meso*-arylporphyrins indicates that the pyrrole NH protons are notably shielded, resulting in their appearance at −2.83 ppm with a low-intensity signal (Figures S7, S9 and S11). Resonance for the β-pyrrolic protons occurs at approximately 9 ppm, in contrast to the phenolic protons, in which resonance is found between 8.9 and 7.2 ppm.

#### 2.2.2. IR Spectroscopy

The IR spectra of the novel *meso*-arylporphyrins **H_2_TNPP**, **H_2_TPPP**, and **H_2_TIPP** are depicted in Figures S13–S15 (see Supporting Information). The IR data for these compounds are collated in Table 1.

Free-base porphyrins are characterized by the presence of three vibrational bands: one pyrrolic υ(NH) vibrational band, one porphyrinic υ(NH) vibrational band, and one porphyrinic δ(CCH) deformational band. For our three *meso*-arylporphyrins, the broad singlet bands in the neighborhood of 3300 cm^−1^ are attributed to the pyrrolic υ(NH) vibrational bands. υ(CH) vibrations, however, are often found as a triplet in the 2978–2884 cm^−1^ range and δ(CCH) warping vibrations often resonate around 966 cm^−1^.

#### 2.2.3. UV–Visible Spectroscopy

The UV–visible spectra of the novel *meso*-arylporphyrins **H_2_TNPP**, **H_2_TPPP**, and **H_2_TIPP** are found in Figure S16 (see Supporting Information). Table 2 summarizes the UV-visible absorption data of several *meso*-aryl porphyrins, including **H_2_TNPP**, **H_2_TPPP**, and **H_2_TIPP**. Our three novel *meso*-arylporphyrins exhibit comparable UV-v–isible spectral characteristics in dichloromethane comprising a Soret band with a maximum absorption wavelength (λ_max_) of around 419 nm, alongside four Q bands at roughly 516, 551, 591, and 647 nm.

The term optical band gap energy (Eg) describes the existing deviation in energy among the HOMO and LUMO orbitals. This parameter is significant in the semiconductor and nanomaterial sectors, and it can be approximated using data obtained from UV–visible spectral analysis. The Eg values for our three new *meso*-arylporphyrins (Figure S17; see Supporting Information) were calculated using a Tauc plot expressed as ${\left(\alpha h\upsilon \right)}^{2}=A\left(h\upsilon -E_{g}\right)$ [31]. Here, A denotes a constant parameter that is influenced by the transition probability, h indicates the energy of the incident photon, and α represents the optical absorption coefficient, which is extracted from the absorbance measurements. These Eg values are 1.875, 1.874, and 1.876 eV for **H_2_TNPP**, **H_2_TPPP**, and **H_2_TIPP**, respectively, thus demonstrating their semiconductor characters.

#### 2.2.4. Fluorescence Spectroscopy

The fluorescence spectra of *meso*-arylporphyrins **H_2_TNPP**, **H_2_TPPP**, and **H_2_TIPP** in dichloromethane are found in Figure S18 (see Supporting Information). Table 2 presents their fluorescence spectral characteristics, as well as those of other *meso*-porphyrins from the literature.

The fluorescence emission spectra (Figure S18) show a strong emission band at 650 nm [Q(0.0)] and a weaker band at 716 nm [Q(0.1)] for **H_2_TNPP**, **H_2_TPPP**, and **H_2_TIPP**. These values are similar to those for the other *meso*-porphyrins from the literature listed in Table 2. The transition, denoted as Q(0,0), is associated with the shift from the second excited singlet state (S2) to the ground state (S0) (i.e., the Soret band), while Q(0,1) corresponds to that from the first excited singlet state (S1) to S0 (i.e., Q bands). As presented in Table 2, the values of the fluorescence quantum yield (Φ_f_) of our new *meso*-arylporphyrins are higher compared to classical porphyrins.

#### 2.2.5. Singlet Oxygen

Singlet oxygen (^1^O_2_) is an activated form of oxygen that is generated in all organisms, particularly photosynthetic ones, and in macrophages and neutrophils in mammals [32,33,34]. The electronic structure of ^1^O_2_ contributes to its classification as a powerful oxidizing agent, capable of causing critical injuries to cellular elements such as DNA, RNA, proteins, and membranes [35]. The ^1^O_2_ luminescence spectra of the three *meso*-arylporphyrins upon excitation at 420 nm in dichloromethane are found in Figure S19 (see Supporting Information). The data are summarized in Table 3.

The ^1^O_2_ formation from our three porphyrins was detected through recording its luminescence, the maximum of which was around 1275 nm after excitation at 420 nm (Figure S19; see Supporting Information). These values, recorded in Table 3, tell us that this type of compound is perfectly suited to photodynamic therapy (PDT) applications due to its high ^1^O_2_ production (^1^O_2_ quantum yields (Φ_Δ_) around 50%).

### 2.3. Anti-Fungal Activity

#### 2.3.1. Anti-Candidal Activity

The anti-fungal activities of our three *meso*-arylporphyrins **H_2_TIPP**, **H_2_TNPP**, and **H_2_TPPP** were quantitatively evaluated using the liquid microdilution method of the international standard of the European Committee on Antimicrobial Susceptibility Testing (EUCAST) using E.Def 7.3.2 against three yeast strains: *C. albicans* (ATCC90028), *C. glabrata* (ATCC 64677), and *C. tropicalis* (CCYT 66029). This method serves to conduct a minimum inhibitory concentration (MIC) values assessment, and the minimum fungicidal concentration (MFC) values were estimated following the inoculation of sensitive wells onto Sabouraud dextrose agar (SDA) plates.

The synthesized compounds were proven to be effective against the three *Candida* strains, with an MIC and MFC ranging from 1.25 to >10 mg/mL and 2.5 to 5 mg/mL, respectively (Table 4).

These results are interesting, especially when compared with previous studies on the same fungal strains; H_2_TTP and [Mg(TTP)] demonstrated minor anti-fungal potential with an MIC and MFC ranging from 5 to 10 mg/mL [37].

A general improvement in the anti-candidal activity seemed to emerge, driven by the relative position effect of the nitrogen atom and ester group on the pyridine ring of **H_2_TIPP**, **H_2_TNPP**, and **H_2_TPPP** (i.e., from *ortho*- to *meta*- to *para*-) (Table 4).

The importance of the steric hindrance and its effect on the potential inhibition of **H_2_TIPP**, **H_2_TNPP**, and **H_2_TPPP** was also proven by comparing the *ortho*-, *meta*-, and *para*-substituted compounds. Obviously, the *ortho*-substituted compound (**H_2_TPPP**) provides more bulk than the *meta*- (**H_2_TNPP**) and *para*- (**H_2_TIPP**)-substituted compounds. Therefore, the presence of groups in the *ortho*-position has a higher negative impact. Therefore, the presence of groups in the *ortho*-position has a higher negative impact. While the *ortho*-isomer’s position contributes to the inhibitory effect, other factors, such as electronic effects or hydrogen bonding, may also influence the observed properties.

#### 2.3.2. Anti-Dermatophyte Activity

**Figure 2:**
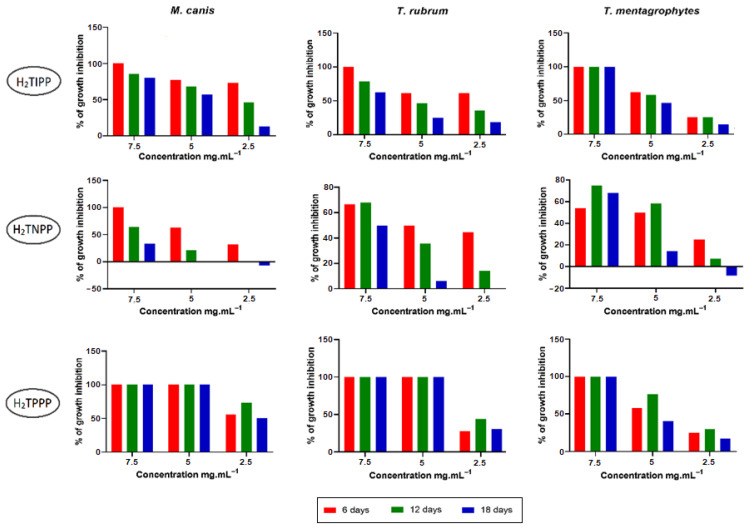
In vitro anti-fungal activity of **H_2_TNPP**, **H_2_TPPP**, and **H_2_TIPP** against the three dermatophyte strains.

The new *meso*-arylporphyrins **H_2_TIPP**, **H_2_TNPP**, and **H_2_TPPP** demonstrated an anti-fungal effect on dermatophyte cultures in a dose-dependent manner. The results of the anti-fungal effects of different dilutions on mycelial growths are shown in Figure 2 and the related micrographs are presented in Figures S20–S22 (see Supporting Information). Porphyrins **H_2_TIPP** and **H_2_TPPP** showed 100% inhibition at a concentration of 7.5 mg/mL on *T. rubrum* and *T. mentagrophytes* strains up to 18 days of cultivation. However, porphyrin **H_2_TNPP** demonstrated an inhibition of about 60% at a concentration of 7.5 mg/mL; this activity persisted over three weeks of culture. The concentration of 5 mg/mL was 100% inhibitory for **H_2_TPPP**, while maintaining its effect for the 18 days of culture, for the *T. rubrum* strain. Nevertheless, other porphyrins (**H_2_TIPP** and **H_2_TNPP)** demonstrated an inhibition of no more than 68% at this concentration, which could drop to 6.66% after 18 days of culture. The concentration of 2.5 mg/mL demonstrated maximum activity against strains *T. rubrum* and *T. mentagrophytes*, not exceeding 45%, and which was not maintained for **H_2_TNPP**, over the following weeks. Nonetheless, lower inhibition percentages were calculated for the remaining porphyrins (**H_2_TIPP** and **H_2_TPPP**) with the same concentration.

Cultures of *M. canis* demonstrated a susceptibility of 100% for up to 5 mg/mL, maintained over 3 weeks of culture, for **H_2_TPPP** porphyrin. An inhibitory effect reaching approximately 100% was noted for both **H_2_TIPP** and **H_2_TNPP** porphyrins at a concentration of 7.5 mg/mL during the first week. Nevertheless, this effect decreased to 80% for **H_2_TIPP** and to 60% for **H_2_TNPP** within 18 days. The 5 mg/mL concentration of **H_2_TIPP** and **H_2_TNPP** inhibited at least 50% of the colony growth for more than 12 days.

In comparison to previous studies from the literature, H_2_TTP and [Mg(TTP)] porphyrins presented low inhibition potential on both *T. rubrum* and *M. canis* dermatophyte strains, not exceeding 20% inhibition at a 5 mg/mL [37].

The variability of activity within the studied fungal species is linked to the availability of drug targets within their cellular structures, such as ergosterol and its biosynthetic enzymes, mainly lanosterol 14α-demethylase or cell wall enzymes like β-glucan synthases [38]. These targets are regulated via gene expression, which varies between species, influencing their susceptibility to anti-fungal agents [39].

### 2.4. Molecular Docking Study

The encouraging anti-fungal properties exhibited by our novel *meso*-arylporphyrins **H_2_TIPP**, **H_2_TNPP**, and **H_2_TPPP** prompted us to undertake molecular docking studies to elucidate their potential mechanisms of action. Within the realm of in silico structure-based drug design, molecular docking emerged as a valuable technique for accurately predicting potential biological targets, particularly in scenarios where experimental enzyme assays are limited. The porphyrin-inhibitory activity was ranked based on the piecewise linear potential fitness (PLP fitness) involved in the process of forming a complex within the active regions of dermatophytes. The determination of the PLP fitness score incorporates the participation of non-covalent interactions such as those among protein–ligand pairs (i.e., hydrogen bond (H-bond) and van der Waals) and those involving only ligand atoms (i.e., intramolecular H-bonds and strains). The interaction capability of a compound is determined by this fitness score, with a greater PLP fitness score indicating a stronger binding affinity [40,41,42]. Only data from *C. tropicalis* and *T. rubrum* matched that of our novel *meso*-arylporphyrins’ **H_2_TIPP**, **H_2_TNPP**, and **H_2_TPPP** structures, on the PDBe.

#### 2.4.1. *Candida tropicalis*

The *C. tropicalis* yeast crystal structure was available on the protein database bank, revealing the extracellular aspartic proteinase (PDBe entry 1j71) [43]. The PLP fitness scores of the docked **H_2_TIPP**, **H_2_TNPP**, and **H_2_TPPP** are given in Table 5.

**Figure 3:**
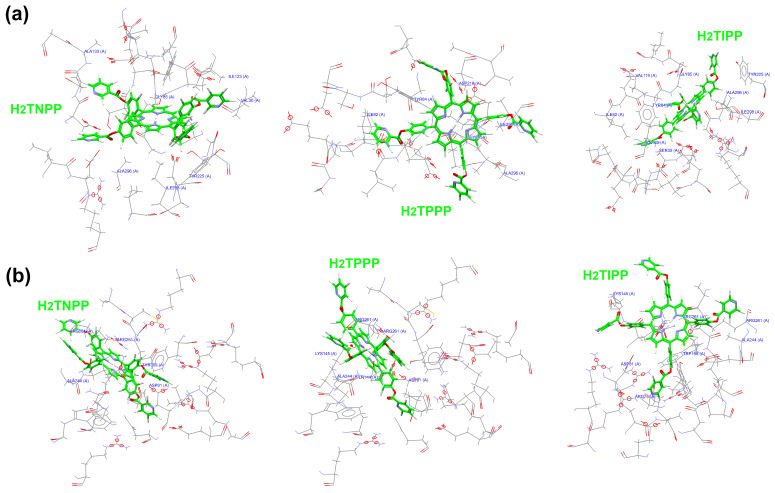
**Three-dimensional** structure image of **H_2_TIPP**, **H_2_TNPP**, and **H_2_TPPP** in anti-fungal complex with *C. tropicalis* (PDBe: 1j71) (**a**) or *T. rubrum* (PDBe: 7P1D) (**b**) protein using Hermes program.

**Figure 4:**
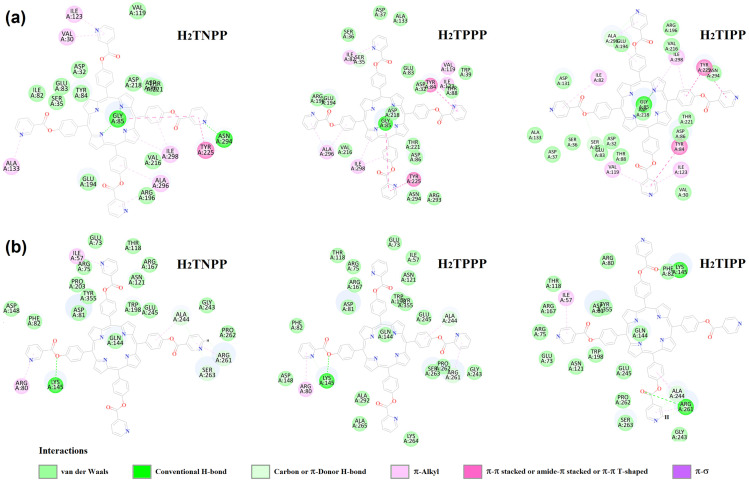
Two-dimensional representation illustrating bond lengths, amino acid residues, and the nature of interactions present with **H_2_TIPP**, **H_2_TNPP**, and **H_2_TPPP** in the active site of *C. tropicalis* (PDBe: 1j71) (**a**) and *T. rubrum* (PDBe: 7P1D) (**b**) using BIOVIA Discovery Studio Visualizer.

As illustrated in Table 5 and Figure 3a and Figure 4a, the carbonyl group of the ester of **H_2_TIPP** establishes an H-bond with the Ser35 (S35) amino acid residue in the active site of the enzyme. The considered pose is reinforced by various intermolecular contacts with the surrounding receptor residues (i.e., π-donor and carbon H-bond with Gly85 (G85) for **H_2_TNPP** and **H_2_TPPP**; π-shaped bond with Tyr225 (Y225) and Tyr84 (Y84) for **H_2_TPPP** and with Tyr225 (Y225) for **H_2_TIPP**). The three *meso*-arylporphyrins form several interactions with the target receptor. Their considered poses are also stabilized by the presence of van der Waals interactions. Extracellular aspartic proteinases have been described as key factors in *Candida* infections and host–pathogen interactions, and Sapt1-Sapt4 have been described for *C. tropicalis* species, with Sapt 2 possibly involved in biofilm formation and its inhibition leading to increased survival in infected animals. Furthermore, an inhibitory effect of non–antifungal drugs (HIV protease inhibitors), namely Ritonavir, on *Candida* spp. extracellular aspartic proteinases (Saps) has been reported [23].

#### 2.4.2. *Trichophyton rubrum*

The *T. rubrum* yeast dermatophyte crystal structure was available on the PDBe entry 7P1D) [44]. The PLP fitness scores of the docked **H_2_TIPP**, **H_2_TNPP**, and **H_2_TPPP** are shown in Table 5.

As illustrated in Table 5 and Figure 3b and Figure 4b, the ester carbonyl group of **H_2_TNPP** and **H_2_TPPP** establishes an H-bond with the Lys145 (K145) amino acid residue in the enzyme’s active site. The considered pose for **H_2_TNPP** and **H_2_TPPP** is reinforced by various intermolecular contacts with the surrounding receptor residues (i.e., π-donor and carbon H-bond with Ala244 (A244), Arg261 (R261), and Ser263 (S263) for **H_2_TNPP** and with Ala244 (A244) and Arg261 (R261) for **H_2_TPPP**; π-alkyl interaction with Arg80 (R80) for **H_2_TNPP** and **H_2_TPPP**). Concerning **H_2_TIPP**, the formation of four interactions with the target receptor should be noted: two H-bonds implying the ester carbonyl group with the Lys145 (K145) and Arg261 (R261) amino acid residues in the enzyme’s active site; the π-donor and carbon H-bond with Ala244 (A244); the π-alkyl interaction with Ile57 (I57). The considered poses of our three *meso*-arylporphyrins are also stabilized by the presence of van der Waals interactions.

The potential target of *T. rubrum* (PDBe: 7P1D) belongs to Sialidases (KDNases). Sialidases are enzymes that facilitate sialic acid liberation at the terminal ends of glycan chains. Evidence suggests that the Sialidase, referred to as KDNase, from *A. fumigatus* is crucial for maintaining cell wall integrity and for its virulence in mice treated with Amphotericin B [45].

## 3. Materials and Methods

### 3.1. Chemicals and Reagents

All chemicals, unless otherwise noted, were acquired with the highest commercially available purity and were used without any subsequent purification.

### 3.2. Materials Instrumentation

The NMR spectra were recorded on a Brucker Advance 300 spectrophotometer. The spectra were recorded in CDCl_3_ and DMSO-*d_6_* at 298 K using solvent residual peaks (CDCl_3_: δ = 7.26 ppm for ^1^H and 77.1 ppm for ^13^C; DMSO-*d_6_*: δ = 2.50 ppm for ^1^H and 39.5 ppm for ^13^C) as an internal reference. Chemical shifts (δ) are expressed in parts per million (ppm), while coupling constants (*J*) are measured in Hertz (Hz). The multiplicity is characterized as s for singlet, d for doublet, dd for doublet of doublet, m for multiplet, H_β_ for β-pyrrolic protons, H_Ph_ for phenyl protons, and H_Pyr_ for pyridine protons.

The determination of melting points (Mp) was conducted using a Buchi M-560 device.

Element analyses were conducted with a Carlo Erba model 1106 microanalyzer (INEOS RAS).

The Fourier-transformed IR spectra were obtained using a PerkinElmer Spectrum Two FT-IR spectrometer.

UV–visible spectra were recorded employing a WinASPECT PLUS device, which was validated for SPECORD PLUS (version 4.2).

The analysis involved electrospray mass spectrometry (ESI-MS) and was conducted utilizing a Shimadzu LCMS-2020 Instrument (Shimadzu, Marne la Vallée, France).

Absorption spectra were performed on a double-beam Shimadzu spectrophotometer, model UV-3600.

Fluorescence spectra were conducted using a Horiba Jobin Yvon Fluorolog-3 model FL3-22 spectrofluorometer employing a cell compartment maintained at 25 °C and a 450 W Xenon lamp as the light source. Fluorescence quantum yield (ϕ_f_) was measured using a tetraphenyl porphyrin (H_2_TPP) solution dissolved in dichloromethane, which acted as the fluorescence reference (ϕ_f_ = 0.20) [26]. The quantum yields of singlet oxygen (^1^O_2_), denoted as ϕ_Δ_, were assessed through the direct measurement of infrared luminescence, utilizing H_2_TPP in dichloromethane as a reference standard (ϕ_f_ = 0.62) [36]. The absorbance levels for the reference and sample solutions were adjusted to around 0.2 at the excitation wavelength.

### 3.3. Aldehydes AL1–AL3

#### 3.3.1. Synthesis

All these aldehydes were prepared via an esterification reaction [17] (Scheme 1). To a solution of pyridinecarboxylic acid derivative (24 mmol, 1.5 equiv), 4-hydroxybenzaldehyde (16 mmol, 1.0 equiv) and *N*,*N*-dimethylaminopyridine (DMAP) (2.4 mmol, 0.15 equiv) in dichloromethane (10 mL) was subjected to a dropwise addition of a dichloromethane solution (15 mL) of *N*,*N*′-dicyclohexylcarbodiimide (DCC) (26 mmol, 1.6 equiv) at 0 °C. The mixture was subsequently stirred for 12 h at room temperature in a nitrogen atmosphere. Afterward, the mixture was filtered, and the solvent was removed via evaporation to obtain a dry solid. The obtained solid was washed with water, then dried under vacuum for 2 h. The crude solid was then recrystallized from a dichloromethane and petroleum ether mixture to afford the desired aldehyde **AL1** (4-formylphenyl nicotinate), **AL2** (4-formylphenyl picolinate) or **AL3** (4-formylphenyl isonicotinate) as white powder [46,47,48].

#### 3.3.2. Characterization

**AL1.** Yield: 74%. Mp: 98–100 °C. Elemental analysis calcd (%) for C_13_H_9_NO_3_: C 68.72, H 3.99, N 6.16; found C 68.93, H 4.13, N 6.39. MS (ESI) (MeOH) for C_13_H_9_NO_3_ [M + H]^+^ calcd 228.0660; found 228.0643. FT-IR (solid neat): $\bar{\nu }$ (cm^−1^) = 3106–2945 [ν(CH aromatic)], 2849–2717 [ν(CH aldehyde)], 1735 [ν(C=O ester)], 1696 [ν(C=O aldehyde)], 1587 [ν(C=N)], 1160 [ν(C-N)], 1216–1201 and 1085–1019 [ν(C-O ester)]. ^1^H NMR (300 MHz, DMSO-*d_6_*), δ (ppm): 10.03 (s, CHO, 1H), 9.27 (s, H_Pyr_, 1H); 8.91 (d, *J* = 4.5 Hz, H_Pyr_, 1H); 8.49 (d, *J* = 8.1 Hz, H_Pyr_, 1H); 8.05 (d, *J* = 8.4 Hz, H_Ph_, 2H); 7.67 (dd, *J* = 7.8 and 5.1 Hz, H_Pyr_, 1H); 7.59 (d, *J* = 8.4 Hz, H_Ph_, 2H). ^13^C NMR (75 MHz, DMSO-*d_6_*), δ (ppm): 192.2 (C=O, aldehyde), 163.3 (C=O, ester), 154.9 (C_Ph_), 154.5 (CH_Pyr_), 150.7 (CH_Pyr_), 137.7 (CH_Pyr_), 134.3 (C_Ph_), 131.2 (2 CH_Ph_), 124.9 (C_Pyr_), 124.2 (CH_Pyr_), 122.9 (2 CH_Ph_).

**AL2.** Yield: 79%. Mp 100–104 °C. Elemental analysis calcd (%) for C_13_H_9_NO_3_: C 68.72, H 3.99, N 6.16; found C 68.95, H 4.16, N 6.38. MS (ESI) (MeOH for C_13_H_9_NO_3_ [M + H]^+^ calcd 228.0660; found 228.0645. FT-IR (solid neat): $\bar{\nu }$ (cm^−1^) = 3104–2929 [ν(CH aromatic)], 2850–2719 [ν(CH aldehyde)], 1742 [ν(C=O ester)], 1690 [ν(C=O aldehyde)], 1599–1563 [ν(C=N)], 1153 [ν(C-N)], 1229–1191 and 1091–1057 [ν(C-O ester)]. ^1^H NMR (300 MHz, DMSO-*d_6_*), δ (ppm): 10.02 (s, CHO, 1H); 8.81 (d, *J* = 4.2 Hz, H_Pyr_, 1H); 8.24 (d, *J* = 7.8 Hz, H_Pyr_, 1H); 8.11–8.01 (m, 1 H_Pyr_ and 2 H_Ph_, 3H); 7.74 (dd, *J* = 7.2 and 4.5 Hz, H_Pyr_, 1H); 7.55 (d, *J* = 8.4 Hz, H_Ph_, 2H). ^13^C NMR (75 MHz, DMSO-*d_6_*), δ (ppm): 192.1 (C=O, aldehyde), 163.0 (C=O, ester), 155.3 (C_Ph_), 150.1 (CH_Pyr_), 146.4 (C_Pyr_), 137.8 (CH_Pyr_), 134.2 (C_Ph_), 131.2 (2 CH_Ph_), 128.2 (CH_Pyr_), 125.9 (CH_Pyr_), 122.8 (2 CH_Ph_).

**AL3.** Yield: 91%. Mp 104–106 °C. Elementary analysis calcd (%) for C_13_H_9_NO_3_: C 68.72, H 3.99, N 6.16; found C 68.97, H 4.14, N 6.41. MS (ESI) (MeOH) for C_13_H_9_NO_3_ [M + H]^+^ calcd 228.0660; found 228.0643. FT-IR (solid neat): $\bar{\nu }$ (cm^−1^) = 3105–2930 [ν(CH aromatic)], 2850–2745 [ν(CH aldehyde)], 1746 [ν(C=O ester)], 1687 [ν(C=O aldehyde)], 1599–1563 [ν(C=N)], 1155 [ν(C-N)], 1217–1191 and 1091–1058 [ν(C-O ester)]. ^1^H NMR (300 MHz, DMSO-*d_6_*), δ (ppm): 10.04 (s, CHO, 1H); 8.90 (d, *J* = 4.5 Hz, H_Pyr_, 2H); 8.08–7.99 (m, 2 H_Pyr_ and 2 H_Ph_, 4H); 7.60 (d, *J* = 7.8 Hz, H_Pyr_, 2H). ^13^C NMR (75 MHz, DMSO-*d_6_*), δ (ppm): 192.1 (C=O, aldehyde), 163.2 (C=O, ester), 154.8 (C_Ph_), 151.0 (2 CH_Pyr_), 136.0 (C_Pyr_), 134.3 (C_Ph_), 131.2 (2 CH_Ph_), 123.0 (2 CH_Pyr_), 122.8 (2 CH_Ph_).

### 3.4. Meso-Arylporphyrins H_2_TNPP, H_2_TPPP and H_2_TIPP

#### 3.4.1. Synthesis

All these new *meso*-arylporphyrins were prepared using the Adler–Longo method [24] (Scheme 2). In a double-necked round-bottom flask, propanoic acid (for **AL1** and **AL3**) or acetic acid (for **AL2**) (100 mL) was used to dissolve aldehyde **AL1**, **AL2** or **AL3** (29 mmol, 1 equiv) and the resulting solution underwent reflux heating (110–115 °C). Afterward, freshly distilled pyrrole (29 mmol, 1 equiv) was introduced in a dropwise manner and the resulting mixture was maintained under reflux for 40 min. After cooling overnight at 4 °C, the mixture was filtered under vacuum to afford the desired **H_2_TNPP** (*meso*-tetrakis [4-(nicotinoyloxy)phenyl]porphyrin), **H_2_TPPP** (*meso*-tetrakis [4-(picolinoyloxy)phenyl]porphyrin) or **H_2_TIPP** (*meso*-tetrakis [4-(isonicotinoyloxy)phenyl]porphyrin) as a purple solid.

#### 3.4.2. Characterization

**H_2_TNPP.** Yield: 13%. Mp: >400 °C. Elementary analysis calcd (%) for C_68_H_42_N_8_O_8_: C 74.31; H 3.85; N 10.19; found C 74.51, H 3.98, N 10.43. MS (ESI) (MeCN) for C_68_H_42_N_8_O_8_ [M + H]^+^ calcd 1099.3203; found 1099.3188. UV-vis (CH_2_Cl_2_) λ/nm (log ε): 419 (5.97), 551 (4.51), 561 (4.06), 591 (3.73), 647 (3.44). FT-IR (solid neat): $\bar{\nu }$ (cm^−1^) = 3310 [ν(NH porphyrin)], 1732 [ν(C=O ester)], 1588 [ν(C=C porphyrin)], 1499–1472 [ν(C=N porphyrin)], 1270 [ν(C-O ester)], 966 [δ(C-CH porphyrin)]. ^1^H NMR (CDCl_3_, 300 MHz), δ (ppm): 9.59 (s, 4H, H_Pyr_), 8.95 (s, 8H, H_β_), 8.72–8.62 (m, 8H, H_Pyr_), 8.32 (d, *J* = 8.4 Hz, 8H, H_Ph_), 7.68 (d, *J* = 8.4 Hz, 8H, H_Ph_), 7.59 (dd, *J* = 7.5 and 4.8 Hz, 8H, H_Ph_), −2.76 (s, 2H, NH). ^13^C NMR (CDCl_3_, 75 MHz), δ (ppm): 164.8 (4 C=O, ester), 154.9 (4 CH_Pyr_), 152.3 (4 CH_Pyr_), 151.3 (4 C_Ph_ and 8 C_α_), 140.7 (4 C_Ph_), 138.5 (4 CH_Pyr_), 136.2 (8 CH_Ph_ and 8 CH_β_), 126.4 (4 C_Pyr_), 124.3 (4 CH_Pyr_), 120.6 (8 CH_Ph_), 119.9 (4 C*_meso_*).

**H_2_TPPP.** Yield: 15%. Mp: >400 °C. Elementary analysis calcd (%) for C_68_H_42_N_8_O_8_: C 74.31, H 3.85, N 10.19; found C 74.54, H 3.92, N 10.38. MS (ESI) (MeCN) for C_68_H_42_N_8_O_8_ [M + H]^+^ calcd 1099.3203; found 1099.3192. UV-vis (CH_2_Cl_2_) λ/nm (log ε): 419 (5.86), 551 (4.40), 561 (3.93), 591 (3.61), 647 (3.32). FT-IR (solid neat): $\bar{\nu }$ (cm^−1^) = 3310 [ν(NH porphyrin)], 1739 [ν(C=O ester)], 1589–1558 [ν(C=C porphyrin)], 1501–1472 [ν(C=N porphyrin)], 1268 [ν(C-O ester)], 965 [δ(C-CH porphyrin)]. ^1^H NMR (DMSO-*d_6_*, 300 MHz), δ (ppm): 9.94 (s, 4H, H_Pyr_), 8.86 (s, 12H, 8 H_β_ and 4 H_Pyr_), 8.00 (d, *J* = 8.4 Hz, 12H, 8 H_Ph_ and 4 H_Pyr_), 7.21 (d, *J* = 8.4 Hz, 12H, 8 H_Ph_ and 4 H_Pyr_), −2.88 (s, 2H, NH). ^13^C NMR (DMSO-*d_6_*, 75 MHz), δ (ppm): 157.6 (C=O, ester), 148.6 (4 CH_Pyr_), 147.2 (4 C_Ph_ and 8 C_α_), 138.4 (4 CH_Pyr_), 135.9 (8 CH_Ph_ and 8 CH_β_), 132.3 (4 C_Ph_), 120.4 (4 C_Pyr_), 115.8 (4 CH_Pyr_), 115.6 (4 CH_Pyr_), 114.4 (8 CH_Ph_), 109.9 (4 C*_meso_*).

**H_2_TIPP.** Yield: 14%. Mp: >400 °C. Elementary analysis calcd (%) for C_68_H_42_N_8_O_8_: C 74.31, H 3.85, N 10.19; found C 74.49, H 3.95, N 10.41. MS (ESI) (MeCN) for C_68_H_42_N_8_O_8_ [M + H]^+^ calcd 1099.3203; found 1099.3186. UV-vis (CH_2_Cl_2_) λ/nm (log ε): 419 (5.75), 551 (4.29), 561 (3.80), 591 (3.48), 647 (3.20). FT-IR (solid neat): $\bar{\nu }$ (cm^−1^) = 3310 [ν(NH porphyrin)], 1743 [ν(C=O ester)], 1601–1563 [ν(C=C porphyrin)], 1501–1472 [ν(C=N porphyrin)], 1266 [ν(C-O ester)], 965 [δ(C-CH porphyrin)]. ^1^H NMR (CDCl_3_, 300 MHz), δ (ppm): 8.99 (d, *J* = 4.8 Hz, 8H, H_Pyr_), 8.94 (s, 8H, H_β_), 8.31 (d, *J* = 8.1 Hz, 8H, H_Ph_), 8.21 (d, *J* = 4.8 Hz, 8H, H_Pyr_), 7.67 (d, *J* = 8.1 Hz, 8H, H_Ph_), -2.75 (s, 2H, NH). ^13^C NMR (CDCl_3_, 75 MHz), δ (ppm): 164.6 (C=O, ester), 151.6 (8 CH_Pyr_), 151.2 (4 C_Ph_ and 8 C_α_), 140.9 (4 C_Pyr_), 137.7 (4 C_Ph_), 136.2 (8 CH_Ph_ and 8 CH_β_), 124.1 (8 CH_Pyr_), 120.5 (8 CH_Ph_), 119.8 (4 C*_meso_*).

### 3.5. Biological Evaluation

An examination was conducted on the anti-fungal efficacy (i.e., anti-candidal and anti-dermatophytic activities) of **H_2_TNPP**, **H_2_TPPP**, and **H_2_TIPP** on yeasts belonging to the *Candida* genus, specifically *C. albicans* (ATCC 90028), *C. glabrata* (ATCC 64677), and *C. tropicalis* (ATCC 66029), alongside three strains of dermatophytes, which included *M. canis* (MS 8972), *T. rubrum* (MS 7793.1), and *T. mentagrophytes* (MS45), isolated from scalp lesions and identified by the expert personnel team at the parasitology and mycology laboratory of the University Hospital Fattouma Bourguiba in Monastir (Tunisia). Mycosic strains were kept at 30 °C, for 48 h in culture on Sabouraud dextrose agar (SDA) plates for *Candida* spp. strains and for one week for dermatophytes, before each anti-fungal assay [49].

#### 3.5.1. Anti-Candidal Activity

The assessment of the minimum inhibitory concentrations (MIC) for the three novel *meso*-porphyrins against fungal strains was conducted following the procedure outlined by Hrichi et al. [49]. This procedure incorporated the microdilution technique using flat bottom 96-well plates in conjunction with the Resazurin viability indicator. Each porphyrin was dissolved in dimethylsulfoxide (DMSO) at a weight-to-volume ratio (100 mg/mL), followed by dilution in RPMI 1640 (PAN-Biotech, Hamburg, Germany) enriched with 2% glucose. In the 96-well plate, the first well of each row received 200 µL of the porphyrin solution (20 mg/mL), while an addition of RPMI 1640 supplemented with 2% glucose (100 µL) was performed in the other wells. After that, a two-fold serial dilution was performed across the first ten consecutive wells, resulting in a range of concentrations from 10 to 78 × 10^−2^ mg/mL. Inoculation of each well was performed using inoculum (90 μL) calibrated to 1–2.5 × 10^5^ CFU/mL, followed by the addition of 10 μL of Resazurin indicator solution (0.005 g of resazurin in 10 mL of distilled water) to achieve a total volume of 200 μL in each well. The experimental setup included a positive and a negative growth control consisting of RPMI 1640 supplemented with 2% glucose and 1% DMSO with or without inoculum, respectively, for each experiment. Each plate was incubated for 24 h at 37 °C, and all experiments were replicated three times. Post incubation, a blue color signified the inhibition of growth, while a pink color reflected the resazurin reduction and subsequent strain viability. The concentration that demonstrated the least growth inhibition was designated the MIC. To evaluate the minimum fungicide concentration (MFC), 10 μL from each blue well was transferred onto an SDA plate and incubated at a suitable temperature. Concentrations that exposed three or less colonies to 99–99.5% dead growth after the ensuing one week of incubation, or that did not produce visible colonies, were recognized as MFC.

#### 3.5.2. Anti-Dermatophyte Activity

An in vitro evaluation of the anti-dermatophytic properties of activity of the three novel *meso*-porphyrins was conducted through the agar incorporation method using Petri dishes with a diameter of 30 mm for MIC90 detection [49]. After dissolving each porphyrin candidate in DMSO at a weight-to-volume ratio, the resulting solutions were further dissolved in SDA medium (1 mL) to achieve a final concentration of 10 mg/mL. A negative control well, which contained no substance, was included to track the dermatophytes’ expansion under standard conditions. Mycelial agar disks measuring 2 mm were excised from a culture of dermatophytes that had been actively growing for 1 week and were placed in the center of each Petri dish. Each experiment was replicated three times and the growth inhibition (*I*) for the porphyrin was determined using the following formula:

$$I\;\left(\%\right)=\left[Growth\;in\;control-\frac{Growth\;in\;sample}{Growth\;in\;control}\right]\times 100$$

### 3.6. Molecular Docking Study

The docking process was executed with the GOLD software (v.5.8.0) [50]. The visualization of the docking results was facilitated through the Hermes program available in the GOLD software (v.1.10.4). The binding sites selected for the docking included protein residues situated within 10 Å of the reference ligand found in the protein’s structural complex. The cavity and active site were identified using the CCDC Superstar tool. The determination of the 10 Å radius active site was performed using a reference protein ligand. In this study, the Chemscore kinase served as a representative model profile and ChemPLP was used to assess the scoring function. Default values for all parameters in the GOLD docking procedure were retained and the resulting solutions were ranked using the piecewise linear potential fitness function (ChemPLP). The interactions between the ligands and the protein residues were estimated through docking results, including the binding mode and free energy, as well as the docked pose. At the end of the docking process, an analysis of the interactions within the receptor–ligand complex was made with BIOVIA Discovery Studio Visualizer (v.17.2.0.16349). These results were then formatted as two-dimensional and three-dimensional structures, which contributed to a clearer visualization and analysis of the interaction dynamics within the ligand–protein complex [51].

## 4. Conclusions

In this study, we synthesized three new *meso*-arylporphyrins: **H_2_TIPP**, **H_2_TNPP**, and **H_2_TPPP**. These porphyrins were characterized using NMR, UV–Visible, fluorescence, and IR spectroscopies, as well as mass spectrometry. Their photophysical characteristics, particularly their ability to produce singlet oxygen (Φ_Δ_ values), highlighted their potential uses as PSs in photodynamic therapy (PDT) applications. The anti-fungal efficacy of these new porphyrins was evaluated in vitro against several pathogenic fungi: three yeasts from Candida genus and three genera of dermatophytes. The new *meso*-arylporphyrins showed encouraging and good fungicidal activities and might be potentially used as lead compounds in the exploration of new anti-fungal drug discoveries. Furthermore, molecular docking studies emphasized significant aspects regarding the binding affinity and interaction mechanism of these new *meso*-arylporphyrins with the active sites of key dermatophyte enzymes. Examining pre-residue interactions can reveal both bonded and non-bonded interactions that influence the binding affinity towards the target. The docking investigation was found to be in good agreement with the experimental anti-fungal results, showing that the porphyrinic core and the nature of the fragment in the *β*-position (ester function and pyridine derivative moieties) are essential to ensure a significant binding interaction with the amino acid residues in the active sites of the enzymes.

## Figures and Tables

**Table 1:** IR spectroscopic data (neat solid) of **H_2_TNPP**, **H_2_TPPP**, and **H_2_TIPP**.

Attribution of Bands	IR Bands (cm^−1^)		
	H_2_TNPP	H_2_TPPP	H_2_TIPP
υ(NH) pyrrolic	3310	3310	3310
υ(CH) porphyrin	3099–2970	3066–2971	3067–2973
υ(C=O) ester	1732	1739	1743
υ(C-O) ester	1270	1268	1266
δ(CCH) porphyrin	966	965	965

**Table 2:** UV–visible absorption and fluorescence emission data of several *meso*-aryl porphyrins, and **H_2_TNPP**, **H_2_TPPP**, and **H_2_TIPP** in dichloromethane.

Compounds	UV–Visible Absorption Data						Fluorescence Emission Data (λ_max_ = 420 nm)			Ref.
	Bands λ_max_ (nm) (log ε)					E_gap_ (eV)	Emission (nm)		ϕ_f_	
	Soret	QIV	QIII	QII	QI		Q(0,0)	Q(0,1)		
**H_2_TPP** ^1^	419 (5.48)	516 (4.70)	551 (4.33)	591 (4.19)	648 (4.17)	1.877	652	715	0.20	[25,26]
**H_2_TTP** ^2^	420 (5.96)	518 (4.82)	553 (4.27)	593 (4.16)	650 (4.09)	1.859	653	720	0.20	[25]
**H_2_TClPP** ^3^	420 (5.49)	516 (4.93)	551 (4.54)	591 (4.48)	649 (4.40)	1.921	650	715	0.13	[25,27]
**H_2_TMPP** ^4^	423 (5.55)	519 (4.38)	557 (4.31)	595 (4.26)	652 (4.20)	1.842	657	724	0.39	[28]
**H_2_TBrPP** ^5^	419 (6.65)	515 (5.31)	549 (4.93)	590 (4.77)	648 (4.68)	-	654	720	0.04	[29]
**H_2_TPBP** ^6^	419 (5.90)	515 (4.46)	551 (4.13)	590 (3.94)	646 (3.84)	-	653	715	0.04	[17,30]
**H_2_TMAPP** ^7^	425 (5.92)	522 (5.67)	550 (4.32)	597 (4.15)	653 (3.98)		655	719	0.05	[18]
**H_2_TNPP**	419 (5.97)	516 (4.51)	551 (4.06)	591 (3.73)	647 (3.44)	1.875	650	716	0.13	t.w. ^8^
**H_2_TPPP**	419 (5.86)	516 (4.40)	551 (3.93)	591 (3.61)	647 (3.32)	1.874	650	716	0.16	t.w. ^8^
**H_2_TIPP**	419 (5.75)	516 (4.29)	551 (3.80)	591 (3.48)	647 (3.20)	1.876	650	716	0.19	t.w. ^8^

^1^ TPP = *meso*-tetraphenylporphyrinate, ^2^ TTP = *meso*-methylphenylporphyrinate, ^3^ TClPP = *meso*-tetra(*para*-chlorophenyl)porphyrinate, ^4^ TMPP = *meso*-tetramethoxyphenylporphyrinate, ^5^ TBrPP = *meso*-tetra(*para*-bromophenyl)porphyrinate, ^6^ TPBP = *meso*-tetra[*p*-(benzoyloxy)phenyl]porphyrinate, ^7^ TMAPP = *meso*-tetra{*p*-[((*p*-methoxyphenyl)acetyl)oxy]phenyl}porphyrinate, ^8^ t.w. = this work.

**Table 3:** ^1^O_2_ luminescence emission data of several *meso*-arylporphyrins, and **H_2_TNPP**, **H_2_TPPP**, and **H_2_TIPP** in dichloromethane.

Compounds	Excitation (nm)	Emission (nm)	ϕ_Δ_	Ref.
**H_2_TPP** ^1^	420	1270	0.62	[36]
**H_2_TTP** ^2^	420	1275	0.60	[25]
**H_2_TClPP** ^3^	420	1275	0.77	[25]
**H_2_TMPP** ^4^	420	1275	0.59	[25]
**H_2_TNPP**	420	1275	0.51	t.w. ^5^
**H_2_TPPP**	420	1275	0.52	t.w. ^5^
**H_2_TIPP**	420	1275	0.53	t.w. ^5^

^1^ TPP = *meso*-tetraphenylporphyrinate, ^2^ TTP = meso-tetra(4-tolyl)porphyrinate, ^3^ TClPP = *meso*-tetra(*p*-chlorophenyl)porphyrinate, ^4^ TMPP = *meso*-tetramethoxyphenylporphyrinate, ^5^ t.w. = this work.

**Table 4:** Minimum inhibitory concentration (MIC) and minimum fungicidal concentration (MFC) values of the three *meso*-arylporphyrins against three *Candida* spp. strains.

Compounds	*C. albicans* (ATCC 90028)		*C. glabrata* (ATCC 64677)		*C. tropicalis* (ATCC 66029)	
	MIC (mg/mL)	MFC (mg/mL)	MIC (mg/mL)	MFC (mg/mL)	MIC (mg/mL)	MFC (mg/mL)
**H_2_TIPP**	1.25	2.5	2.5	>10	5	5
**H_2_TNPP**	5	5	5	>10	>10	-
**H_2_TPPP**	2.5	2.5	1.25	2.5	-	-

(-): No inhibition.

**Table 5:** The binding energies for docked **H_2_TIPP**, **H_2_TNPP**, and **H_2_TPPP** with the *C. tropicalis* (PDBe: 1j71) or *T. rubrum* (PDBe: 7P1D) protein complex.

Compounds	*C. tropicalis*		*T. rubrum*	
	Binding Energy (PLP Fitness)	H-Bond and Short Contact Interactions	Binding Energy (PLP Fitness)	H-Bond and Short Contact Interactions
**H_2_TNPP**	146.578	G85-Y225-I123-V30-I298 A296-A133	96.761	K145-R261-A244-S263-R80
**H_2_TPPP**	141.545	G85-Y84-Y225-I298-A296	89.578	K145-R261-A244-R80
**H_2_TIPP**	148.964	G85-Y225-A296-I123-Y84 V119-I82-S35-I298	88.6343	K145-R261-A244-I57

G = Glycine (Gly); Y = Tyrosine (Tyr); I = Isoleucine (Ile); V = Valine (Val); A = Alanine (Ala); S = Serine (Ser); K = Lysine (Lys); R = Arginine (Arg).

## Data Availability

The original contributions presented in this study are included in the article/Supplementary Materials. Further inquiries can be directed to the corresponding authors.

## Supplementary Materials

The following supporting information can be downloaded at: https://www.mdpi.com/article/10.3390/ijms26051991/s1.
