# Supplementary material for: Synthesis, Physicochemical Properties and Anti-Fungal Activities of New Meso-Arylporphyrins
Source: Int J Mol Sci. 2025 Feb 25;26(5):1991. doi: 10.3390/ijms26051991 (PMC11900167; doi:10.3390/ijms26051991)
Supplement: Supplementary file 1 [file ijms-26-01991-s001.zip › ijms-3473841-supplementary.pdf]

Synthesis, Physicochemical Properties and Anti-Fungal Activities of New *Meso*-Arylporphyrins

Hayfa Mkacher [1,2], Raja Chaâbane-Banaoues [3], Soukaina Hrichi [3], Philippe Arnoux [4], Hamouda Babba [3], Céline Frochot [4], Habib Nasri [1,*] and Samir Acherar [2,*]

[1] Laboratory of Physical Chemistry of Materials, Faculty of Science of Monastir, University of Monastir,
Avenue of Environment, Monastir 5019, Tunisia; mkacherhayfa@gmail.com (H.M.); soukaina.hrichi@gmail.com (S.H.)
[2] Université de Lorraine, CNRS, LCPM, F-54000 Nancy, France
[3] Laboratory of Medical and Molecular Parasitology-Mycology (LP3M), Faculty of Pharmacy, University of Monastir, LR12ES08, Monastir 5000, Tunisia; rajachaabanebanaoues@gmail.com (R.C.-B.); hamouda.babba@ms.tn (H.B.)
[4] Université de Lorraine, CNRS, LRGP, F-54000 Nancy, France; philippe.arnoux@univ-lorraine.fr (P.A.); celine.frochot@univ-lorraine.fr (C.F.)
* Correspondence: hnasri1@gmail.com or habib.nasri@fsm.rnu.tn (H.N.); samir.acherar@univ-lorraine.fr (S.A.)

Contents

1. ^{1}H and ^{13}C-JMOD NMR spectroscopy 2

2. IR spectroscopy 11

3. UV-Visible absorption spectroscopy 13

4. Fluorescence emission spectroscopy 14

5. Singlet oxygen luminescence spectroscopy 14

6. Anti-dermatophyte activity 15

1. ^{1}H and ^{13}C-JMOD NMR spectroscopy

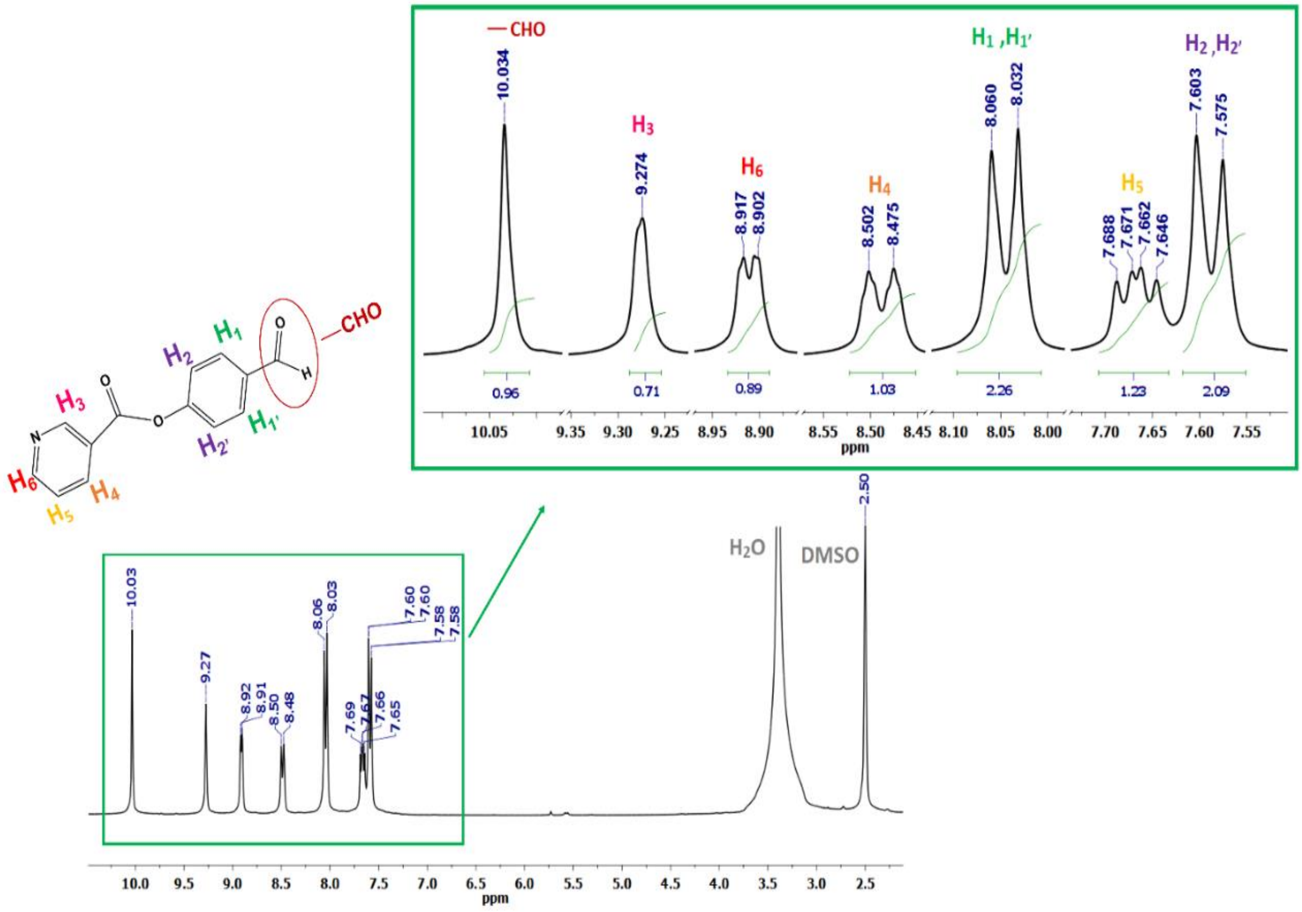

Figure S1. ^{1}H NMR spectrum of **AL1** in the range 2.25–10.25 ppm. Spectrum recorded at room temperature (DMSO-d_6, 300 MHz).

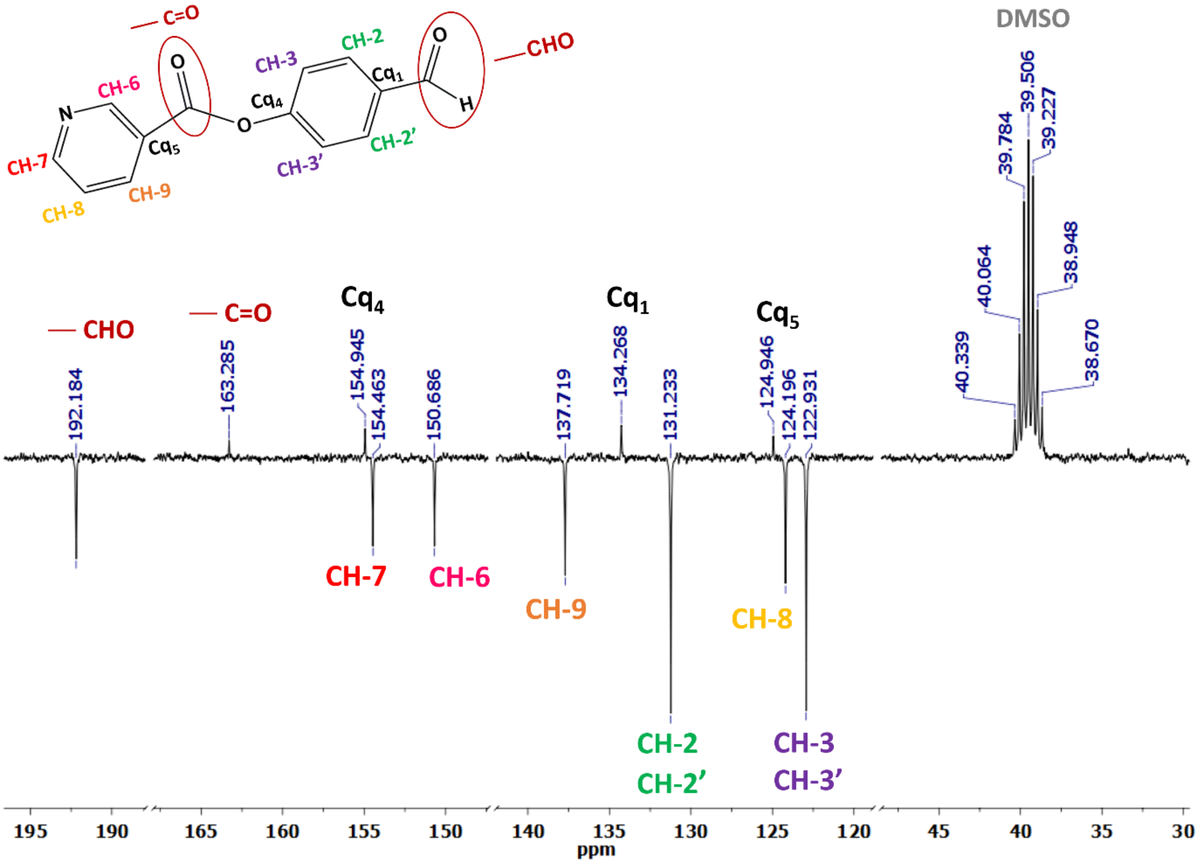

Figure S2. ^{13}C-JMOD NMR spectrum of **AL1** in the range 30–195 ppm. Spectrum recorded at room temperature (DMSO-d_6, 75 MHz).

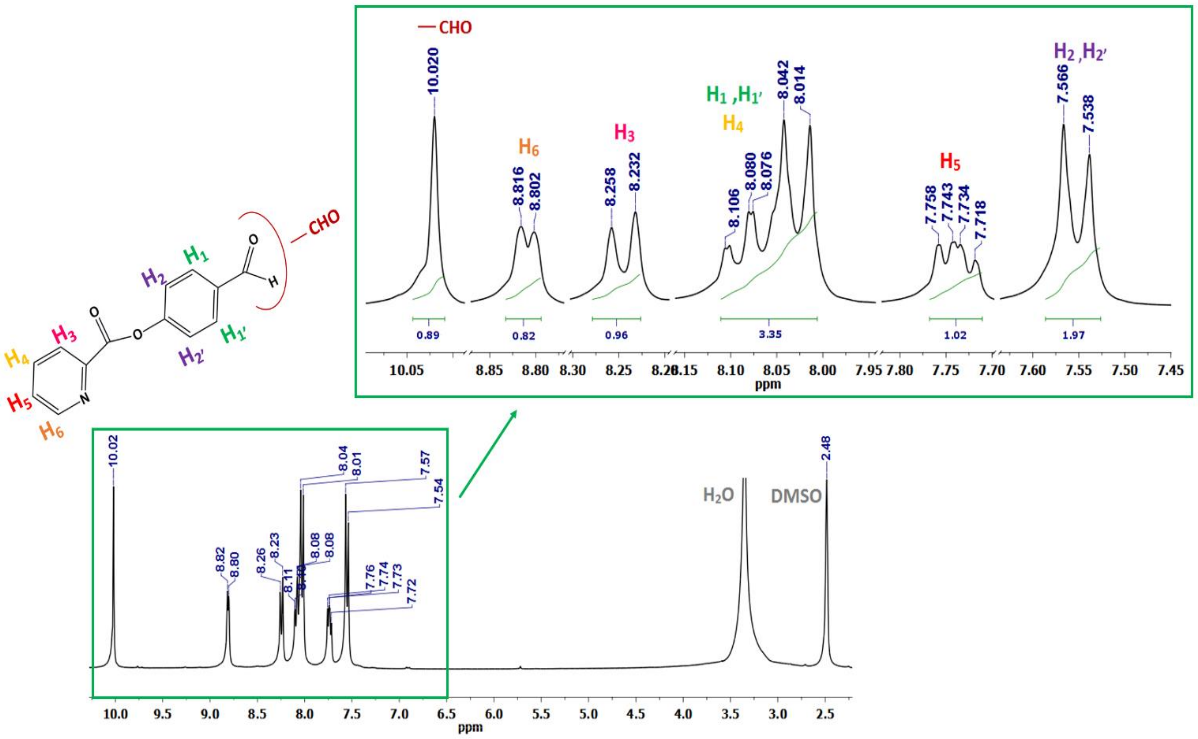

Figure S3. ^{1}H NMR spectrum of **AL2** in the range 2.25–10.25 ppm. Spectrum recorded at room temperature (DMSO-d_6, 300 MHz).

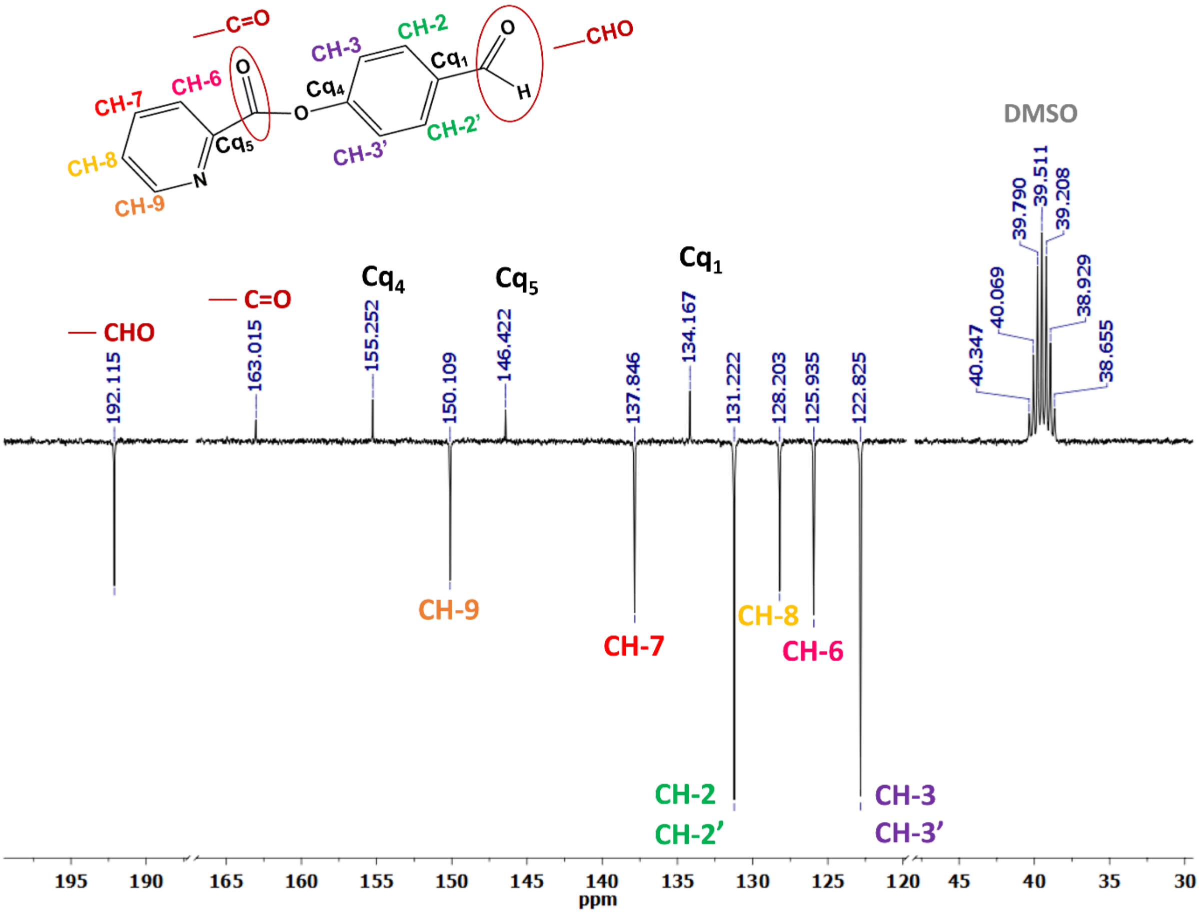

Figure S4. ^{13}C-JMOD NMR spectrum **AL2** in the range 30–195 ppm. Spectrum recorded at room temperature (DMSO-d_6, 75 MHz).

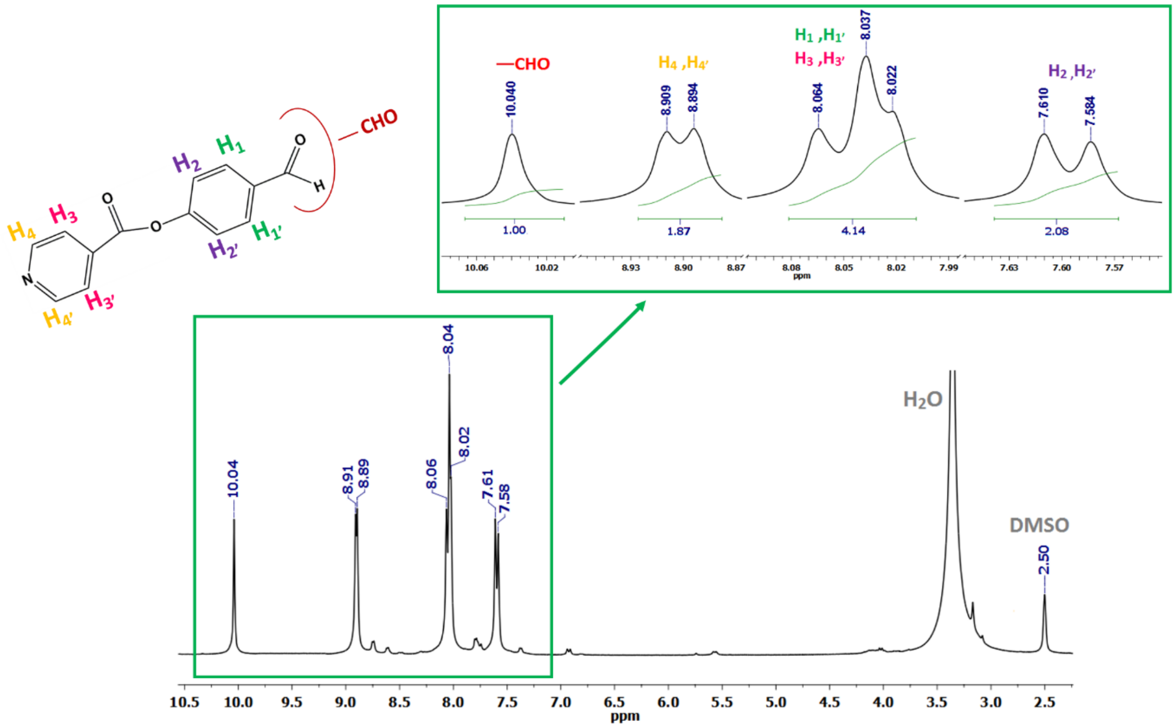

Figure S5. ^{1}H NMR spectrum of **AL3** in the range 2.25–10.25 ppm. Spectrum recorded at room temperature (DMSO-d_6, 300 MHz).

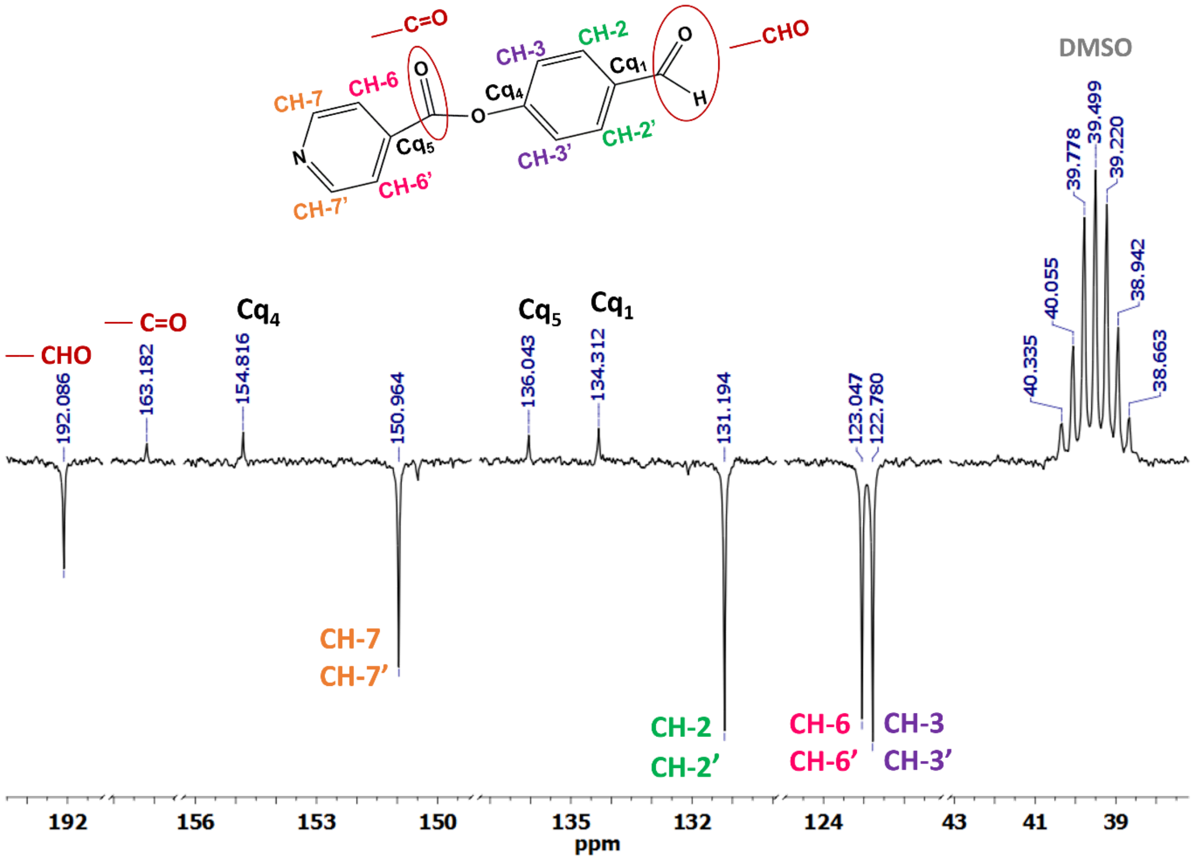

Figure S6. ^{13}C-JMOD NMR spectrum of **AL3** in the range 30–195 ppm. Spectrum recorded at room temperature (DMSO-d_6, 75 MHz).

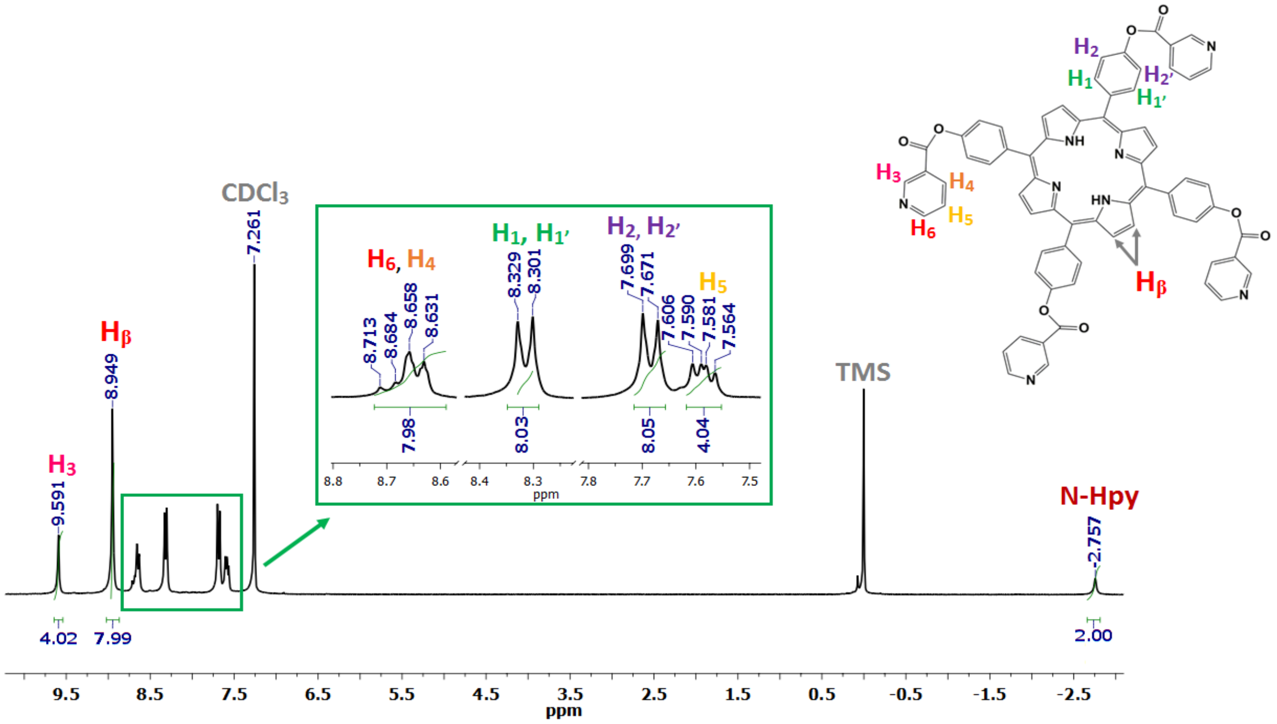

Figure S7. ^{1}H NMR spectrum of **H_2TNPP** in the range -2.25–10.00 ppm. Spectrum recorded at room temperature ($CDCl_3$, 300 MHz).

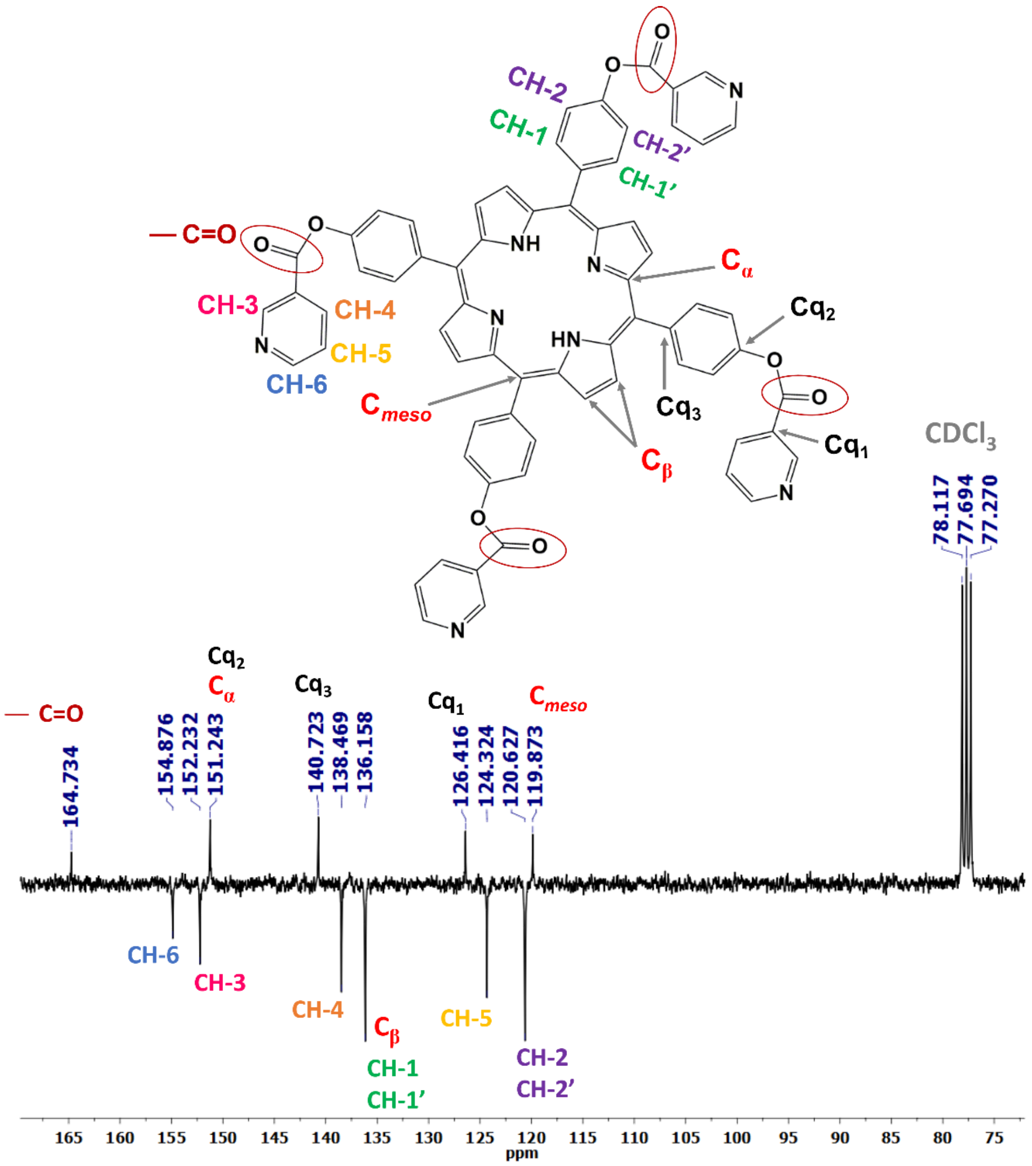

Figure S8. ^{13}C-JMOD NMR spectrum of **H_2TNPP** in the range 75–168 ppm. Spectrum recorded at room temperature ($CDCl_3$, 75 MHz).

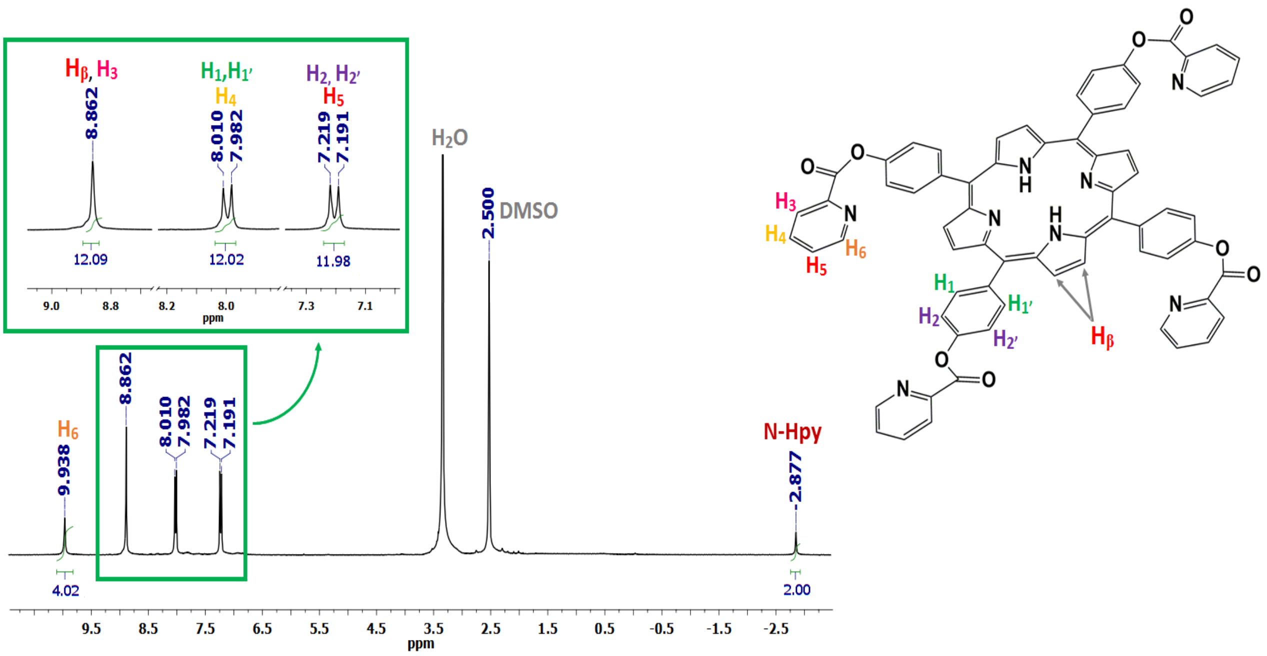

Figure S9. ^{1}H NMR spectrum of **H$_2$TPPP** in the range -2.25–10.50 ppm. Spectrum recorded at room temperature (DMSO-d_6, 75 MHz).

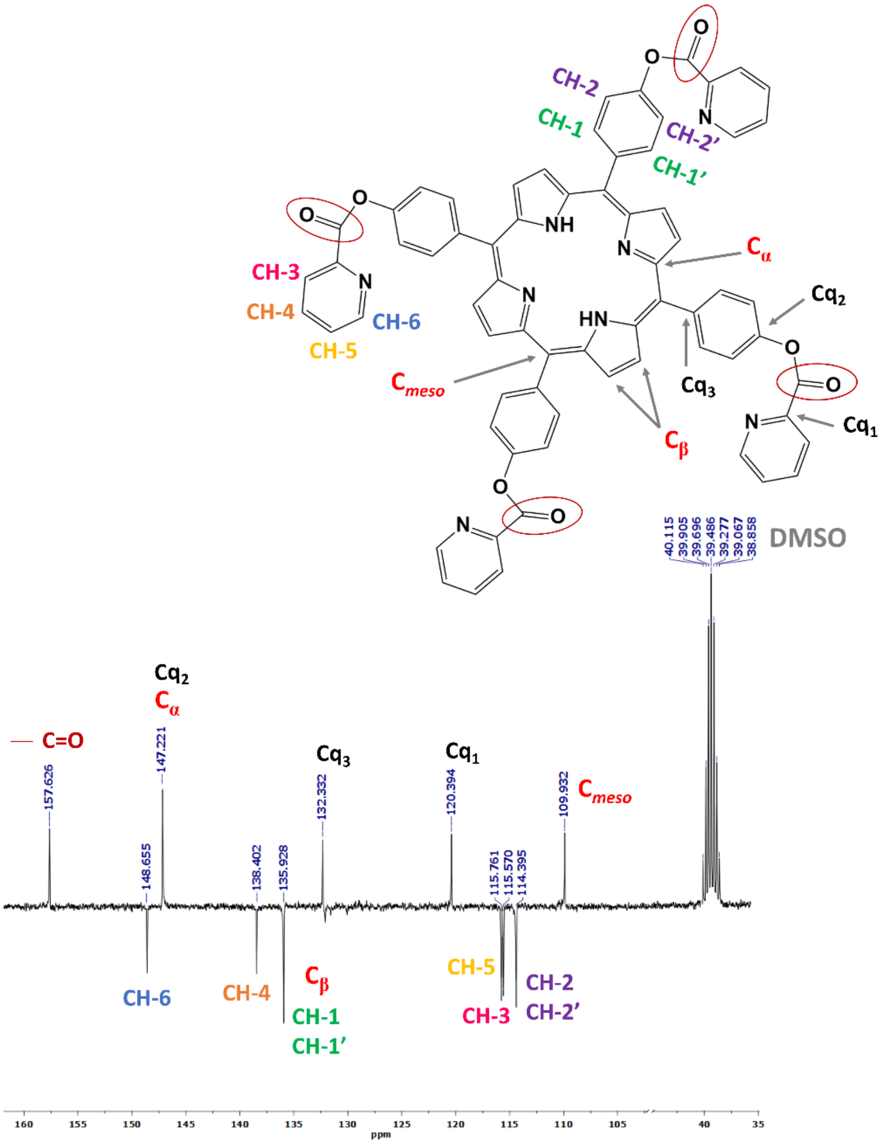

Figure S10. ^{13}C-JMOD NMR spectrum of **H$_2$TPPP** in the range 30–165 ppm. Spectrum recorded at room temperature (DMSO-d_6, 75 MHz).

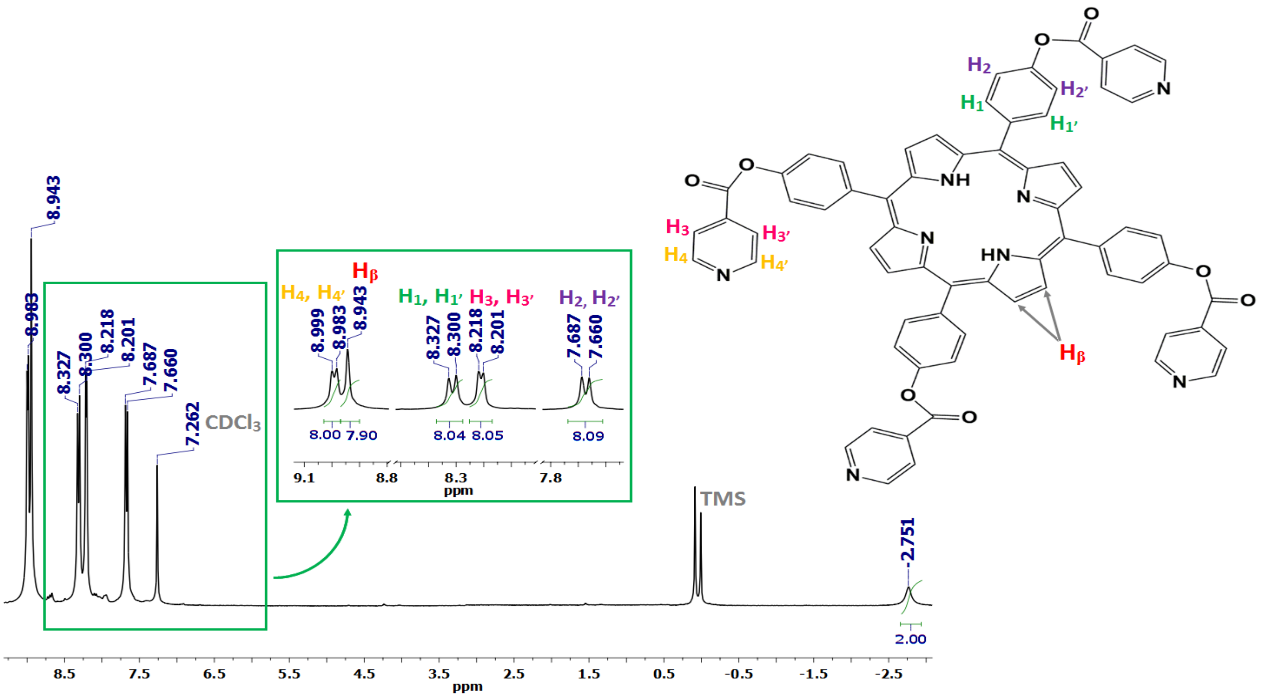

Figure S11. ^{1}H NMR spectrum of **H_2TIPP** in the range -3.00–9.00 ppm. Spectrum recorded at room temperature ($CDCl_3$, 75 MHz).

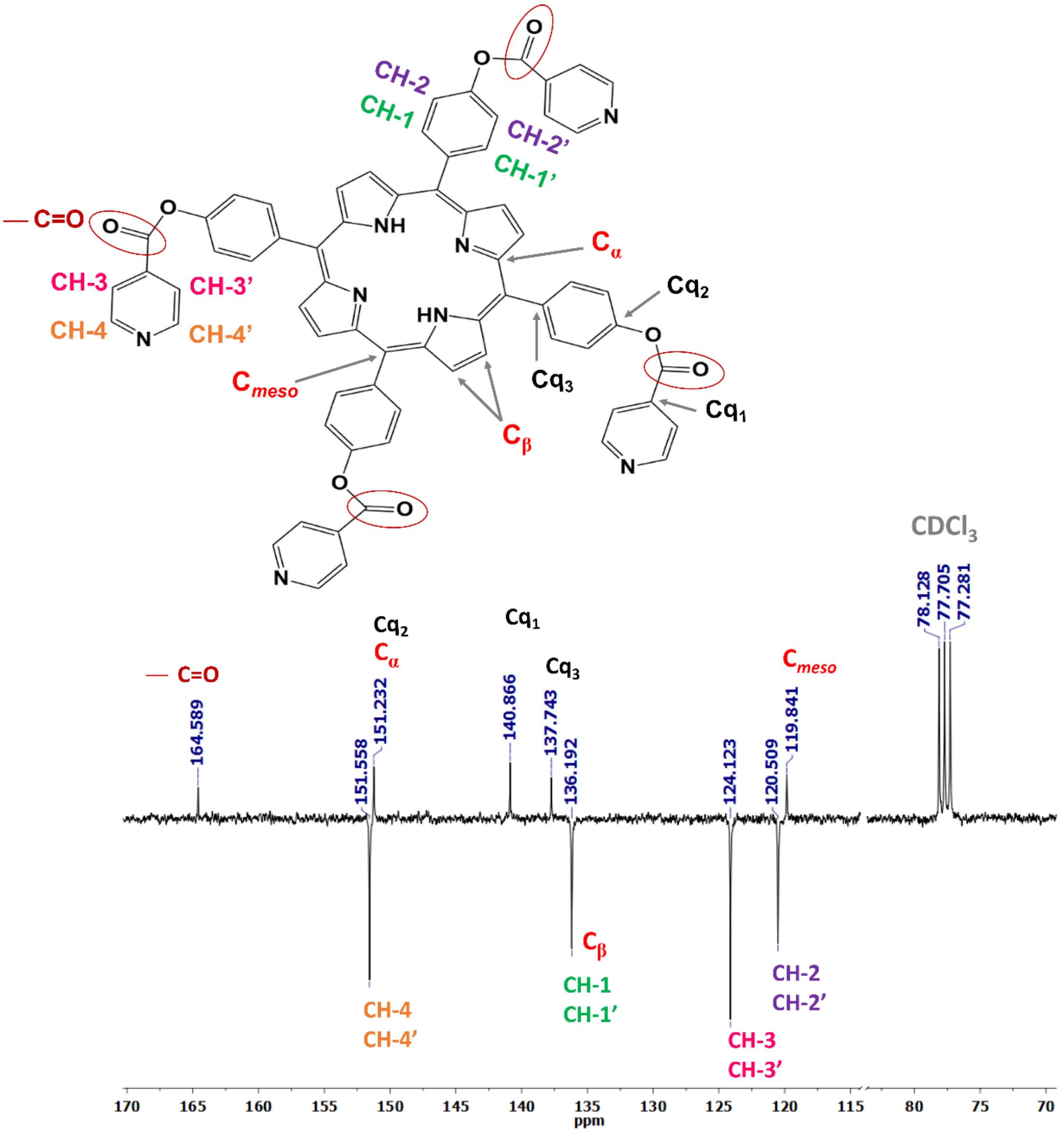

Figure S12. ^{13}C-JMOD NMR spectrum of **H_2TIPP** in the range 75–170 ppm. Spectrum recorded at room temperature ($CDCl_3$, 75 MHz).

2. IR spectroscopy

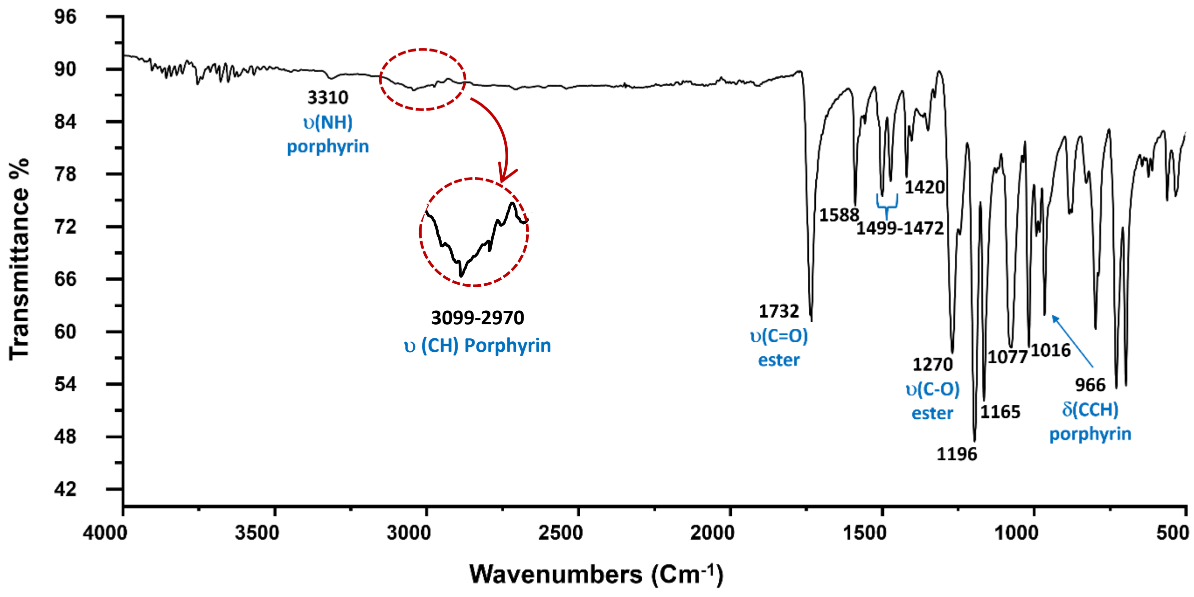

Figure S13. IR spectrum (neat solid) of **H_2TNPP**.

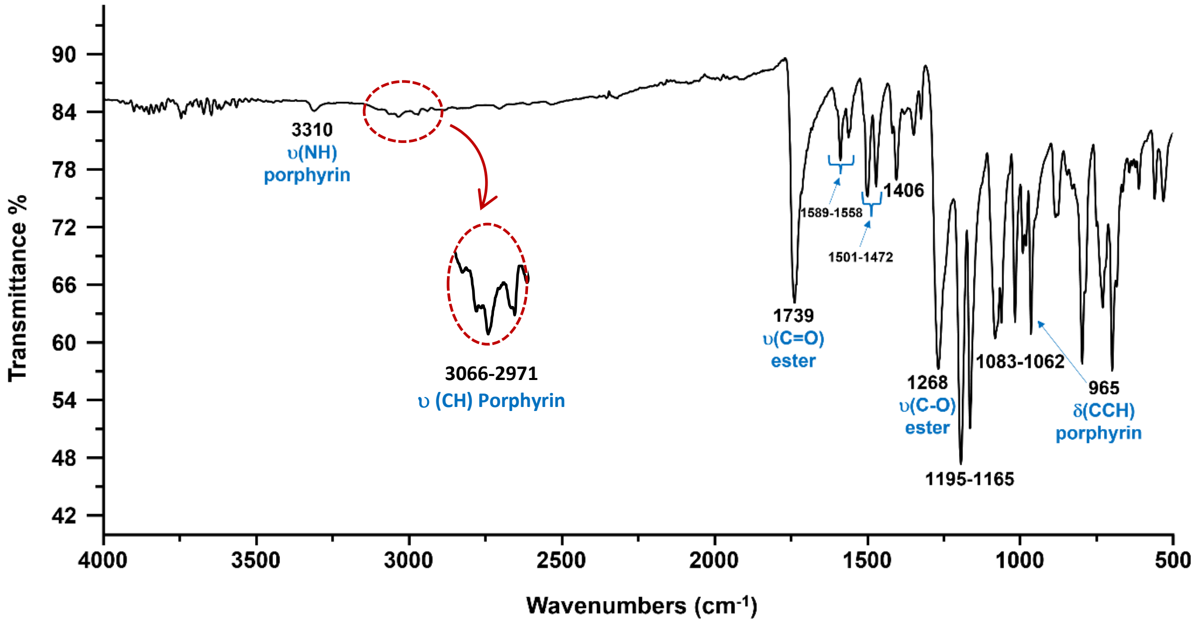

Figure S14. IR spectrum (neat solid) of **H_2TPPP**.

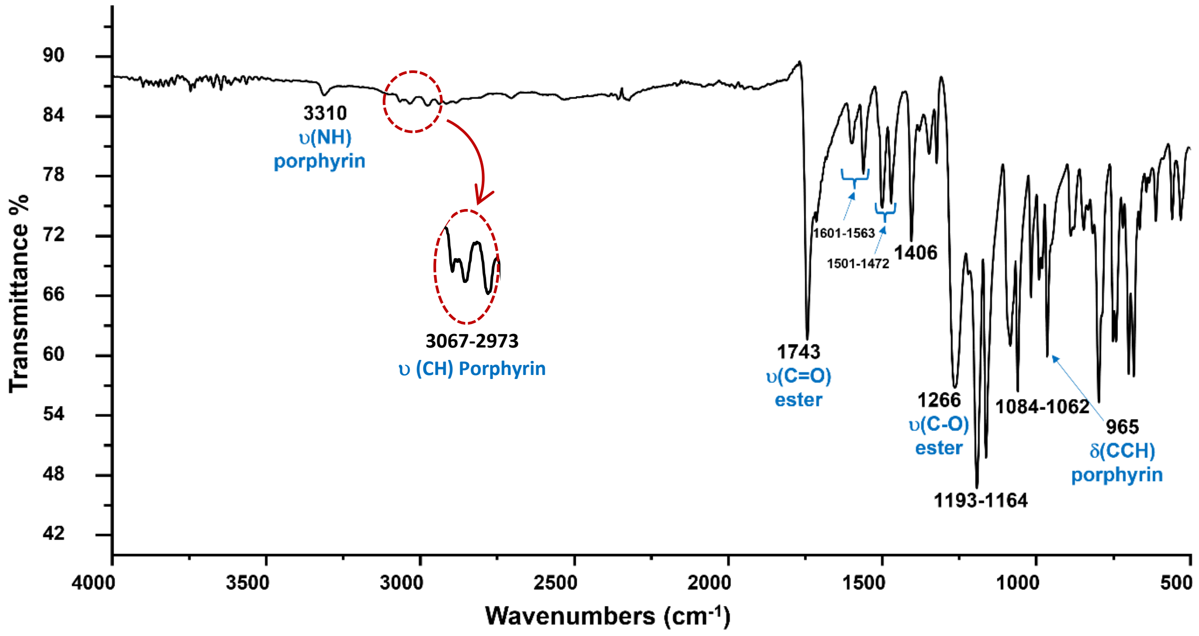

Figure S15. IR spectrum (neat solid) of **H_2TIPP**.

3. UV-Visible absorption spectroscopy

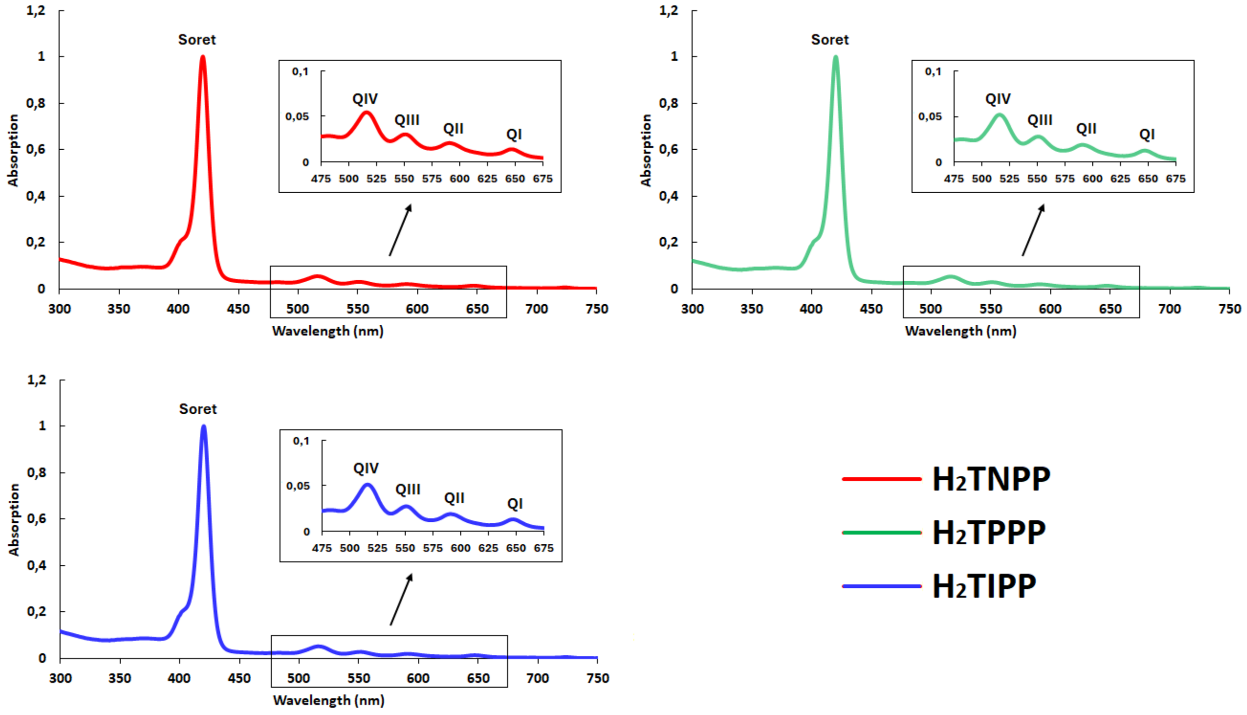

Figure S16. UV-visible absorption spectra of **H_2TNPP**, **H_2TPPP** and **H_2TIPP** (c = 134 µM) (λ_{exc} = 420 nm) in dichloromethane.

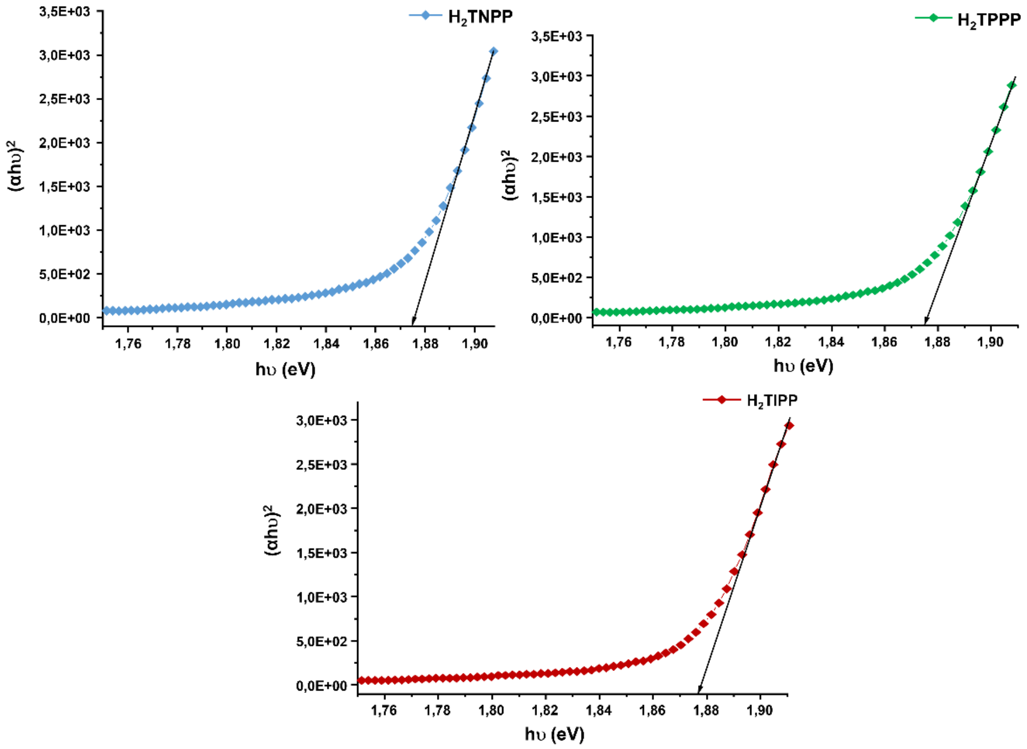

Figure S17. Plots of $(\alpha h\upsilon)^2$ versus the photon energy ($h\upsilon$) of **H_2TNPP**, **H_2TPPP** and **H_2TIPP**. $h\upsilon$ and α are the incident photon energy and the absorption coefficient, respectively.

4. Fluorescence emission spectroscopy

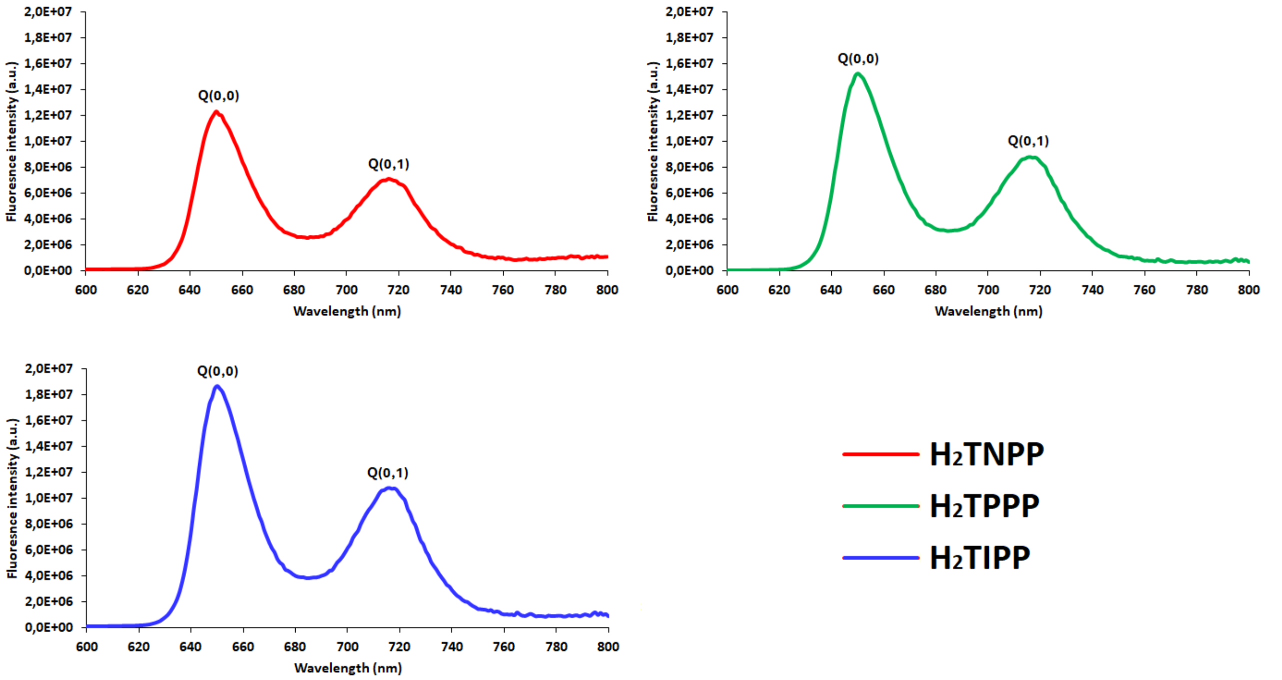

Figure S18. Fluorescence emission spectra of **H_2TNPP** (c = 25 µM), **H_2TPPP** (c = 45 µM) and **H_2TIPP** (c = 56 µM) (λ_{exc} = 420 nm) in dichloromethane.

5. Singlet oxygen luminescence spectroscopy

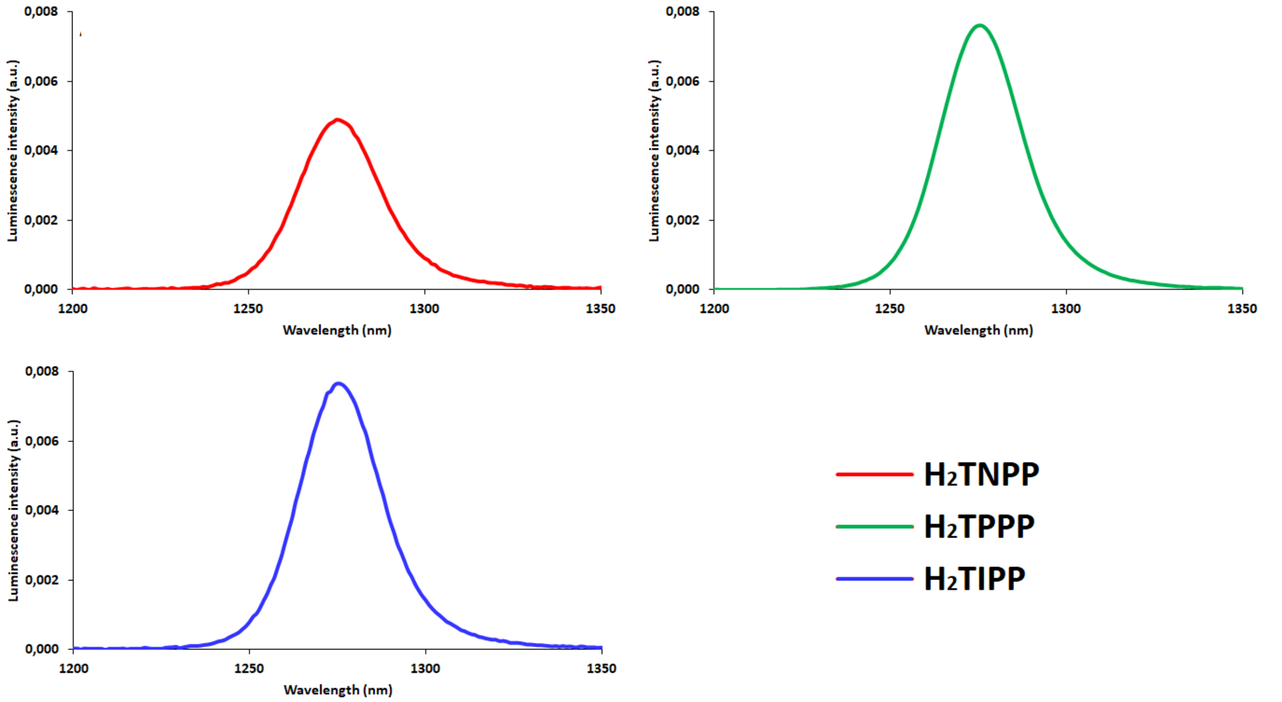

Figure S19. $^1O_2$ luminescence emission spectra of **H_2TNPP** (c = 25 µM), **H_2TPPP** (c = 45 µM) and **H_2TIPP** (c = 56 µM) (λ_{exc} = 420 nm) in dichloromethane.

6. Anti-dermatophyte activity

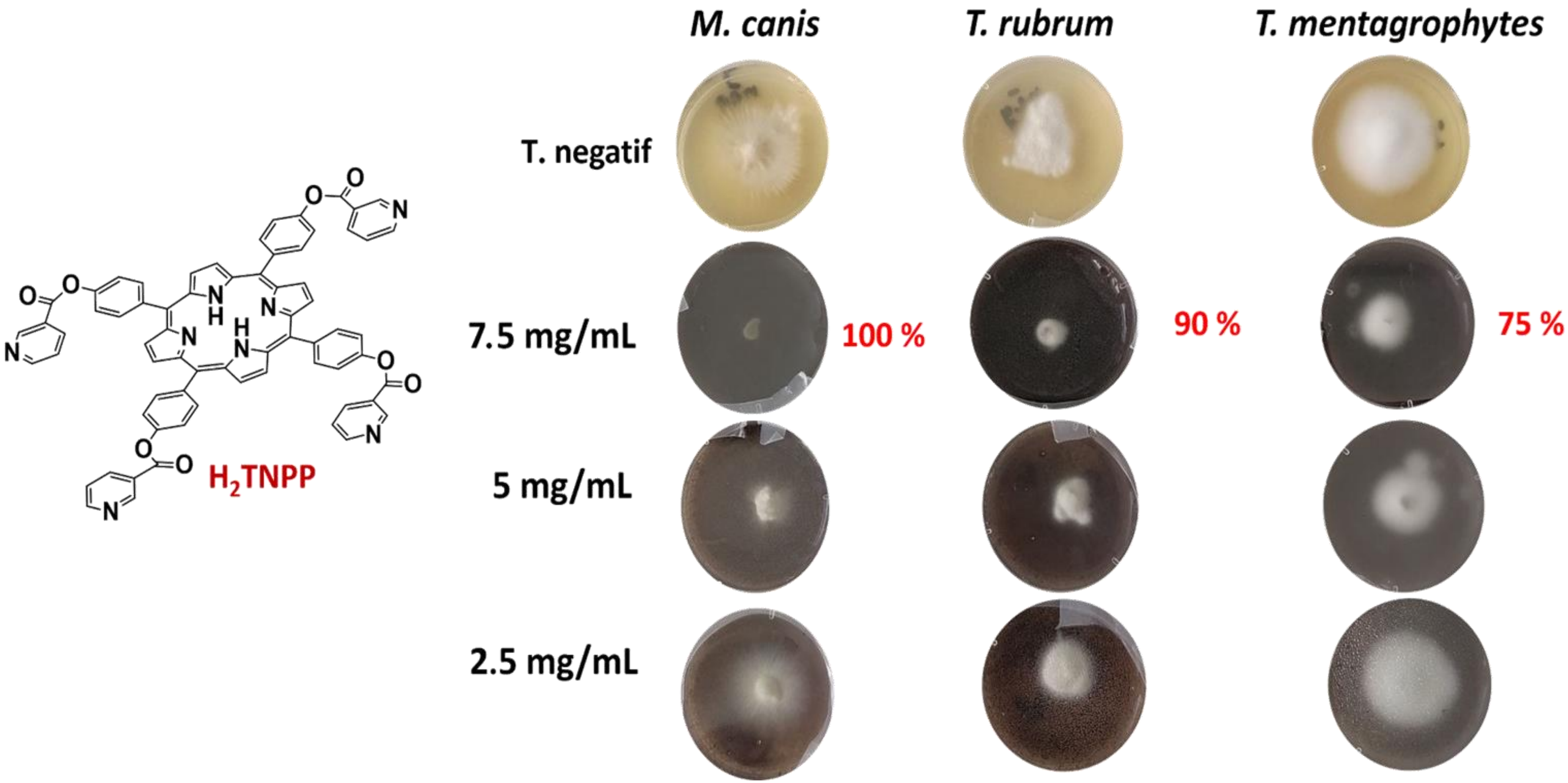

Figure S20. Micrographs of strains of dermatophytes exposed to different concentrations of **H_2TNPP**.

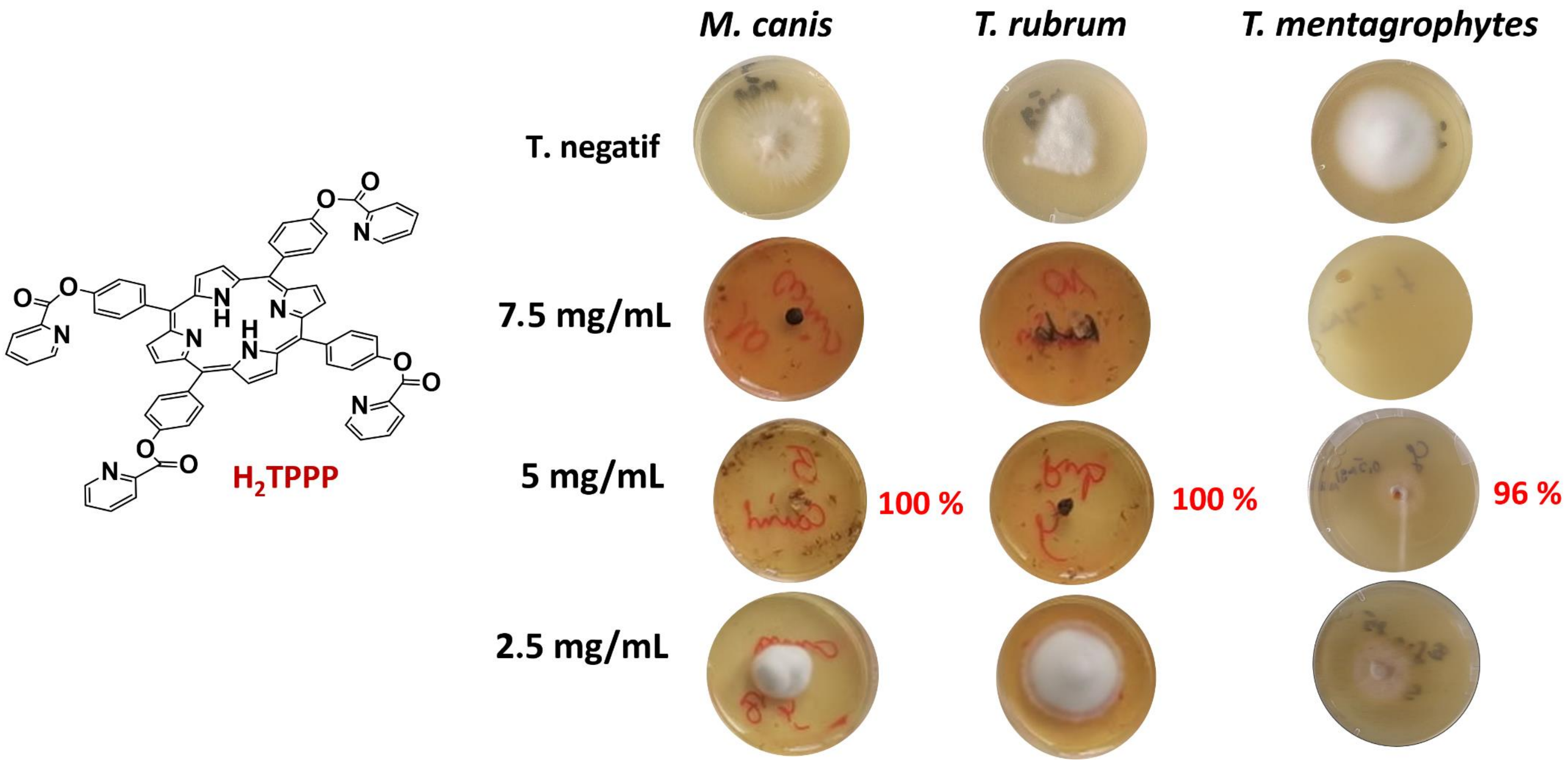

Figure S21. Micrographs of three strains of dermatophytes exposed to different concentrations of **H_2TPPP**.

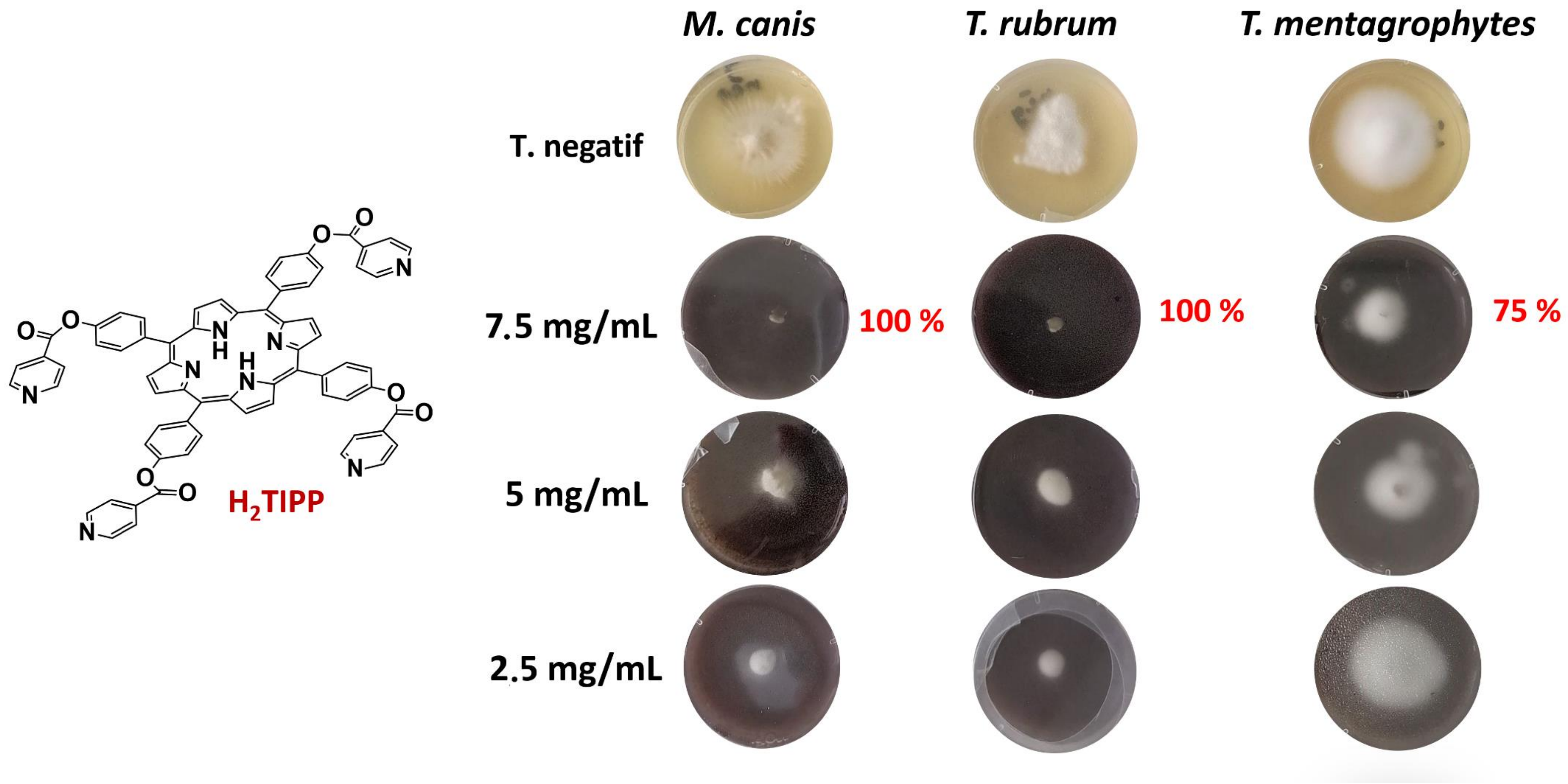

Figure S22. Micrographs of three strains of dermatophytes exposed to different concentrations of **H_2TIPP**.
